# Severe Acute Respiratory Syndrome Coronavirus Envelope Protein Regulates Cell Stress Response and Apoptosis

**DOI:** 10.1371/journal.ppat.1002315

**Published:** 2011-10-20

**Authors:** Marta L. DeDiego, Jose L. Nieto-Torres, Jose M. Jiménez-Guardeño, Jose A. Regla-Nava, Enrique Álvarez, Juan Carlos Oliveros, Jincun Zhao, Craig Fett, Stanley Perlman, Luis Enjuanes

**Affiliations:** 1 Department of Molecular and Cell Biology, Centro Nacional de Biotecnología (CNB-CSIC), Campus Universidad Autónoma de Madrid, Madrid, Spain; 2 Genomics Unit, Centro Nacional de Biotecnología (CNB-CSIC), Campus Universidad Autónoma de Madrid, Madrid, Spain; 3 Department of Microbiology, University of Iowa, Iowa City, Iowa, United States of America; Kantonal Hospital St. Gallen, Switzerland

## Abstract

Severe acute respiratory syndrome virus (SARS-CoV) that lacks the envelope (E) gene (rSARS-CoV-ΔE) is attenuated *in vivo*. To identify factors that contribute to rSARS-CoV-ΔE attenuation, gene expression in cells infected by SARS-CoV with or without E gene was compared. Twenty-five stress response genes were preferentially upregulated during infection in the absence of the E gene. In addition, genes involved in signal transduction, transcription, cell metabolism, immunoregulation, inflammation, apoptosis and cell cycle and differentiation were differentially regulated in cells infected with rSARS-CoV with or without the E gene. Administration of E protein in trans reduced the stress response in cells infected with rSARS-CoV-ΔE or with respiratory syncytial virus, or treated with drugs, such as tunicamycin and thapsigargin that elicit cell stress by different mechanisms. In addition, SARS-CoV E protein down-regulated the signaling pathway inositol-requiring enzyme 1 (IRE-1) of the unfolded protein response, but not the PKR-like ER kinase (PERK) or activating transcription factor 6 (ATF-6) pathways, and reduced cell apoptosis. Overall, the activation of the IRE-1 pathway was not able to restore cell homeostasis, and apoptosis was induced probably as a measure to protect the host by limiting virus production and dissemination. The expression of proinflammatory cytokines was reduced in rSARS-CoV-ΔE-infected cells compared to rSARS-CoV-infected cells, suggesting that the increase in stress responses and the reduction of inflammation in the absence of the E gene contributed to the attenuation of rSARS-CoV-ΔE.

## Author Summary

To identify potential mechanisms mediating the *in vivo* attenuation of SARS-CoV lacking the E gene (rSARS-CoV-ΔE), the effect of the presence of the E gene on host gene expression was studied. In rSARS-CoV-ΔE-infected cells, the expression of at least 25 stress response genes was preferentially upregulated, compared to cells infected with rSARS-CoV. E protein supplied in trans reversed the increase in stress response genes observed in cells infected with rSARS-CoV-ΔE or with respiratory syncytial virus, and by treatment with drugs causing stress by different mechanisms. Furthermore, in the presence of the E protein a subset (IRE-1 pathway), but not two others (PERK and ATF-6), of the unfolded protein response was also reduced. Nevertheless, the activation of the unfolded protein response to control cell homeostasis was not sufficient to alleviate cell stress, and an increase in cell apoptosis in cells infected with the virus lacking E protein was observed. This apoptotic response was probably induced to protect the host by limiting virus production and dissemination. In cells infected with rSARS-CoV-ΔE, genes associated with the proinflammatory pathway were down-regulated compared to cells infected with virus expressing E protein, supporting the idea that a reduction in inflammation was also relevant in the attenuation of the virus deletion mutant.

## Introduction

Severe acute respiratory syndrome coronavirus (SARS-CoV) was identified as the etiological agent of a respiratory disease that emerged in Guandong Province, China at the end of 2002, and spread to 32 countries in a few months [1], [2], [3], [4], [5], [6], [7]. SARS-CoV infected 8000 people in 2002–2003, with an average mortality of 10%. After July 2003, only a few community and laboratory-acquired cases have been reported (http://www.who.int/csr/sars/en/). Nevertheless, coronaviruses similar to the one that caused the epidemic are widely disseminated in bats circulating all over the world, making a future outbreak possible [8], [9], [10].

SARS-CoV is an enveloped, single-stranded positive sense RNA virus, with a genome of 29.7 kb. The coronavirus replicase gene is encoded within the 5′ two thirds of the genome, and includes two overlapping open reading frames (ORFs) named ORF1a and ORF1b. Translation of both ORFs in the cytoplasm of infected cells results in the synthesis of two large polyproteins, pp1b and pp1ab, processed by two viral proteases to yield 16 non structural proteins (nsps) [11], [12]. The nsps are involved in genome replication and transcription of subgenomic mRNAs (sg mRNAs) that encode structural proteins such as the nucleocapsid (N), envelope (E), membrane (M), and spike (S), and a set of group-specific proteins whose sequence and number differ among the different coronavirus species [13]. In the case of SARS-CoV, the group-specific proteins 3a, 6, 7a and 7b, are also structural proteins [14], [15], [16], [17], [18].

SARS-CoV E protein, a small integral membrane protein of 76 amino acids, contains a short hydrophilic amino-terminus followed by a hydrophobic region and a hydrophilic carboxy-terminus [19]. The hydrophobic region forms at least one amphipathic α-helix that oligomerizes to form an ion-conductive pore in membranes [19]. Furthermore, HCoV-229E, murine hepatitis virus (MHV), SARS-CoV, and infectious bronchitis virus (IBV) E proteins form ion channels permeable to monovalent cations [20], [21], [22]. The E protein from genus α transmissible gastroenteritis coronavirus (TGEV) is essential for the generation of propagation competent viruses [23], [24], [25]. In contrast, genus β MHV and SARS-CoV E proteins are not completely essential for the generation of infectious viruses [26], [27], [28]. SARS-CoV lacking the E protein is attenuated in different animal models for SARS, such as hamsters and transgenic mice that express the SARS-CoV receptor, human angiotensin converting enzyme 2 (hACE-2) [26], [27].

Virus infection may result in the expression of stress proteins, like heat shock proteins (hsps), glucose-regulated proteins (GRPs) and ubiquitin [29]. Some of these proteins are constitutively expressed, while others are induced by proteotoxic stresses such as protein overload, heat shock, hypoxia, ischemia, heavy metals, radiation, calcium increase, reactive oxygen species, and drugs, in addition to virus infection [30]. Stress proteins may act as molecular chaperones, participating in protein synthesis, folding, transport, cell viability [31], and modulating the immune response [32]. Increasing evidence suggests that certain hsps play a role in both innate and adaptive immunity [32], [33]. Hsps can act independently of chaperoned peptides to directly stimulate innate immune responses, such as the maturation and activation of dendritic cells, and the activation of natural killer cells (reviewed in [33]).

Coronavirus infection generates double membrane vesicles [34], [35] derived from the endoplasmic reticulum (ER), in which the RNA virus genome is replicated and transcribed [36]. In addition, enveloped viruses modify and perturb membranes to generate new virus particles. This extensive use of intracellular membranes for virus replication and morphogenesis likely overloads the ER during infection, causing ER stress responses and triggering the unfolded protein response (UPR). The UPR increases the production of chaperones that facilitate protein folding, promotes the synthesis of lipids that constitute cellular membranes and inhibits translation in order to reduce ER stress [37]. The UPR is mediated by three ER-resident transmembrane proteins that are activated through binding to unfolded proteins: PKR-like ER kinase (PERK), activating transcription factor 6 (ATF6), and inositol-requiring enzyme 1 (IRE-1) [38], [39], [40]. Upon activation, PERK dimerizes and autophosphorylates. This protein phosphorylates eIF2α, leading to the inhibition of translation. ATF6 activation involves the translocation of this protein to the Golgi compartment, where site 1 and site 2 proteases process the 90 KDa form to create a 50 KDa form, the ATF6α(C), a soluble transcription factor that translocates to the nucleus and upregulates the expression of genes involved in protein folding. IRE-1 mediates the splicing of the mRNA encoding the transcription factor X box-binding protein 1 (XBP-1), leading to a frame shift and translation of a functional XBP-1 protein. The active transcription factor (sXBP-1) can then stimulate the transcription of genes encoding proteins that promote the folding, transport, and degradation of ER proteins, and lipid biosynthesis.

The ER stress response acts to restore ER homeostasis. However, when homeostasis cannot be restored, persistent or intense ER stress can also trigger programmed cell death or apoptosis [41], a physiological mechanism to control the number of cells during development and to respond to infections. Autopsy studies have revealed signs of apoptosis in SARS-CoV-infected tissues from patients, such as lung, spleen and thyroid [42], [43]. Accordingly, it has been shown that the infection by SARS-CoV triggers apoptosis in cell cultures via protein kinase R (PKR) [44] and that at least eight SARS-CoV-encoded proteins induce apoptosis [45].

The expression of genes leading to hyperinflammation has been associated with SARS-CoV-induced pathology. In fact, highly elevated expression of inflammatory mediators such as interleukin (IL)-1, -6, and -8, CXCL10/interferon-inducible protein (IP)-10, CCL2/monocyte chemoattractant protein (MCP)-1, CCL5/regulated on activation, normal T expressed and secreted (RANTES), and CXCL9/monokine induced by interferon gamma (MIG), has been described within the circulation and lungs of SARS patients [46], [47], [48], [49], [50], [51].

In this study, the effect of SARS-CoV E protein on host cell responses during virus infection was analyzed for the first time by comparing the transcriptomes of rSARS-CoV-ΔE and rSARS-CoV-infected cells using microarrays and quantitative reverse transcription polymerase chain reaction (qRT-PCR). We showed that SARS-CoV E protein influenced the expression of genes associated to stress response, immunoregulation, inflammation, apoptosis, and cell cycle and differentiation. Among these changes, the effect on stress response was most robust, based on both the number of differentially expressed genes regulating this activity and on the extent of the changes observed. This downregulation of the stress response in the presence of gene E was specific as this process was reversed by providing E protein in trans. In addition, we showed that E protein reduced the cellular stress caused by another respiratory virus, respiratory syncytial virus (RSV), and two drugs (tunicamycin and thapsigargin) that induce stress by different mechanisms. Furthermore, the presence of E protein reduced the activation of the IRE-1 mediated pathway during the UPR. However, the activation of these signaling pathways in the absence of E protein was not sufficient to reverse the cellular stress induced by rSARS-CoV-ΔE since infected cells underwent apoptosis. In addition, the absence of E protein increased the expression of the double specificity phosphatases (DUSP)-1 and DUSP-10, and down regulated proinflammatory cytokines such as CCL2 and CXCL2. Therefore, the effect of E protein on the stress response, including the UPR, and on proinflammatory cytokine expression may explain the attenuation of rSARS-CoV-ΔE *in vivo*.

## Results

### Growth kinetics of SARS-CoV in Vero E6 and MA-104 cells

**Figure 1:**
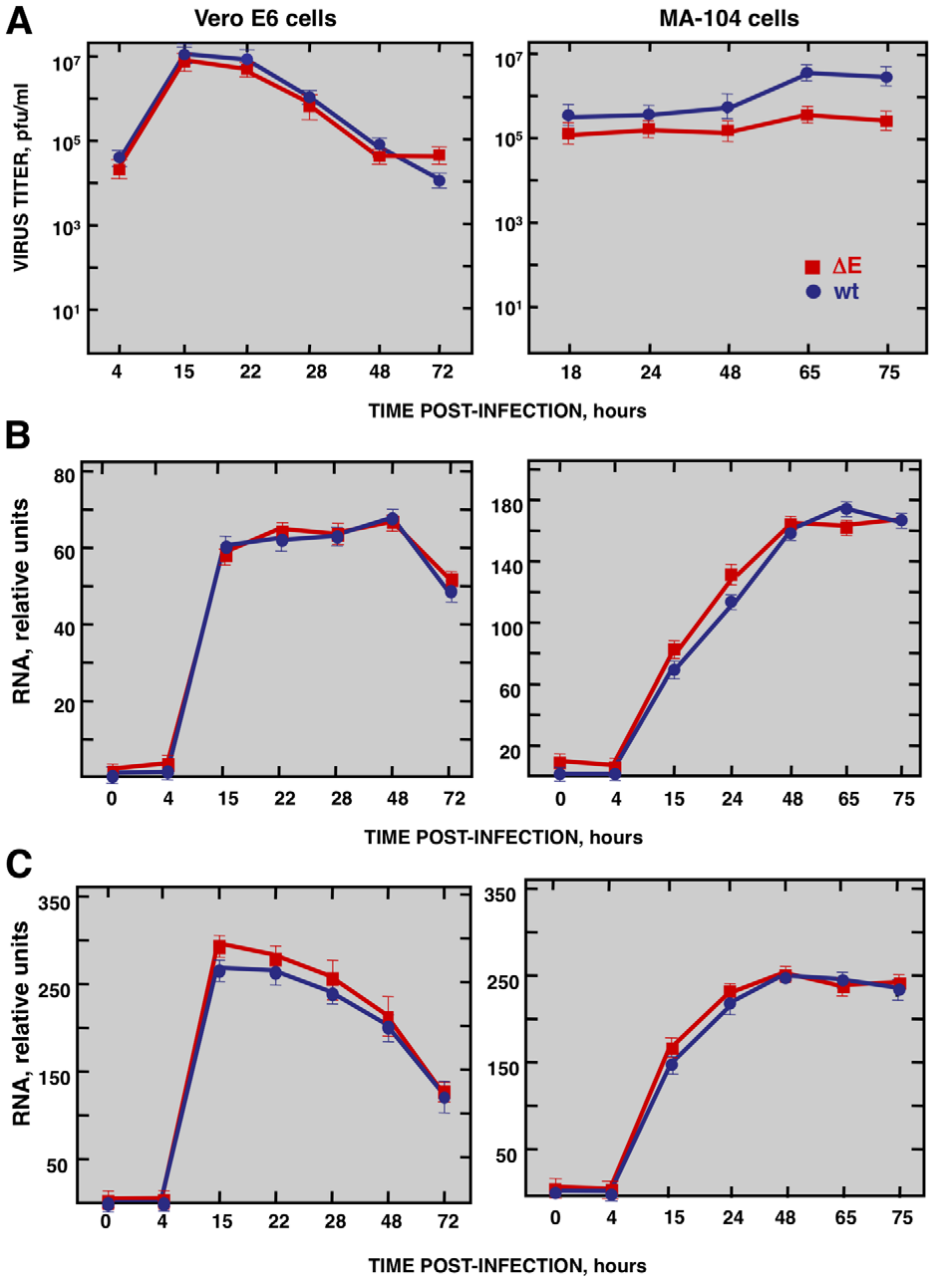
Characterization of infection of Vero E6 and MA-104 cells with rSARS-CoV and rSARS-CoV-ΔE. Vero E6 and MA-104 cells were infected at an moi of 2 with rSARS-CoV-ΔE or rSARS-CoV. (A) Growth kinetics curves. Virus titers in supernatants of infected cells at different times pi were determined by plaque assay. (B) Levels of intracellular genomic RNA in infected cells at different times pi as determined by qRT-PCR. (C) Levels of intracellular N gene sg mRNA in infected cells at different times pi as determined by qRT-PCR. Standard bars represent standard deviations of the mean of results from three experiments.

To study the host response elicited by SARS-CoV, it is essential to use cell lines, such as Vero E6, MA-104, CaCo-2, Huh7, FRhK-4, PK15, HepG2, 293 and 293T cells, that are highly susceptible to infection with SARS-CoV [52], [53], . To determine whether these cell lines were also susceptible to rSARS-CoV-ΔE, virus growth kinetics studies were performed. rSARS-CoV-ΔE passaged 16 times in Vero E6 cells (P16) was analyzed, as this virus grew with titers similar to those of rSARS-CoV, around 10-fold higher than virus passaged only once (P1). rSARS-CoV-ΔE-P16 contained only a single nucleotide substitution at amino acid 607 of the S gene (S607F) compared to the P1 virus [56]. Both rSARS-CoV-ΔE P1 and the P16 were attenuated in the highly susceptible transgenic mice model [56], showing that the deletion of the E gene, and not the amino acid substitution in S protein, was responsible for virus attenuation. All the cell lines indicated above were infected with SARS-CoV with and without E gene at a multiplicity of infection (moi) of 1, 3 and 5 and the percentage of infected cells at 24 hours post infection (hpi) was determined using an immunofluorescence assay. Similar results were obtained both in SARS-CoV and rSARS-CoV-ΔE-infected cells, so only the results obtained with SARS-CoV-infected cells are provided in Supplementary Table SI. An increase in the moi led to a higher proportion of infected cells in all cell lines. The percentage of infected cells was below 40% in all cases, except for African green monkey kidney Vero E6 and MA-104 cells (Supplementary Table S1), which have or do not have, respectively, a defect in interferon (IFN) production [57], [58]. More than 90% of Vero E6 cells were infected with rSARS-CoV-ΔE or rSARS-CoV at 24 hpi, whereas in the case of MA-104 cells, more than 80% of the cells were infected with both viruses at 24 hpi (Supplementary Table S1). The growth kinetics of rSARS-CoV-ΔE and rSARS-CoV in Vero E6 cells at an moi of 2 showed similar profiles and titers for both viruses, reaching maximum titers and cytopathicity at 15 hpi (Fig. 1A). In contrast, in the case of MA-104 cells, although growth kinetics for rSARS-CoV-ΔE and the parental virus were similar, a 10-fold reduction in virus titers was observed in cells infected with rSARS-CoV-ΔE virus (Fig. 1A). This difference is not unexpected, as the ΔE virus that was used in these experiments was passaged and adapted to growth in Vero E6, but not in MA-104 cells. The cytopathic effect in MA-104 cells was evident at 48 hpi and maximum virus titers were reached at 65 hpi (Fig. 1A). The kinetics of genomic RNA and N gene sg mRNA accumulation were similar in rSARS-CoV-ΔE and rSARS-CoV-infected Vero E6 and MA-104 cells, as determined by qRT-PCR (Fig. 1B and 1C), indicating that SARS-CoV E protein had no influence on the accumulation of viral RNAs. Maximum levels of both types of viral RNA were observed at 15–22 hpi, in the case of infected Vero E6 cells and at 48 hpi in the case of MA-104 cells. These data showed that although Vero E6 and MA-104 cells were susceptible to SARS-CoV, the kinetics of the infection was slower in MA-104 than in Vero E6 cells, which needs to be considered when cellular mRNAs are collected for differential gene expression studies.

### Effect of SARS-CoV E protein on host gene expression

**Figure 2:**
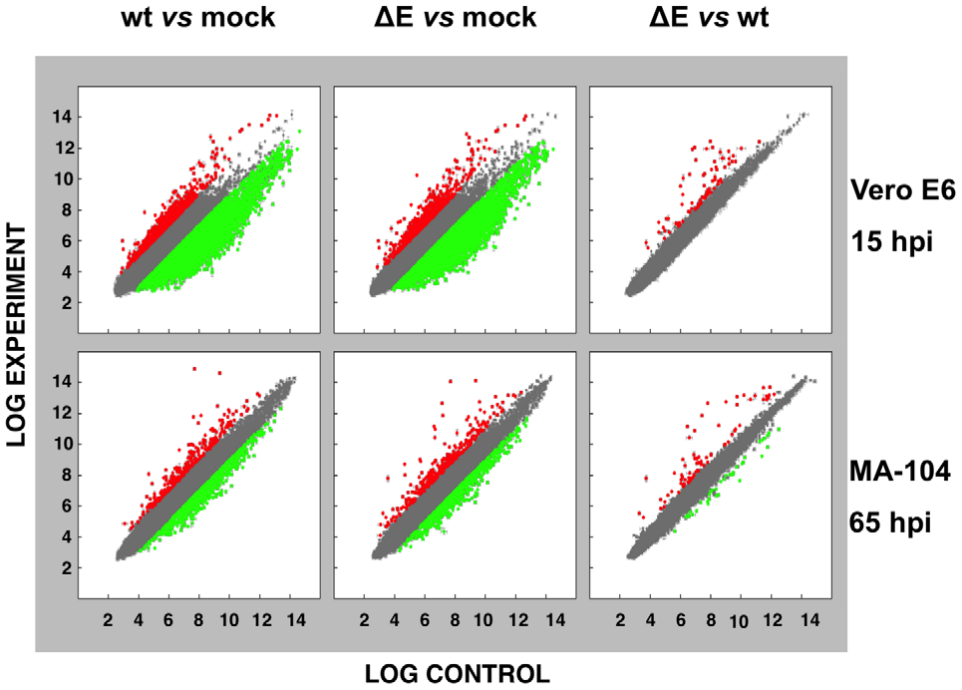
Effect of SARS-CoV E protein on host gene expression. Comparison of gene expression in Vero E6 (at 15 hpi) and MA-104 (at 65 hpi) cells using microarrays: rSARS-CoV versus mock-infected, rSARS-CoV-ΔE versus mock-infected and rSARS-CoV-ΔE versus rSARS-CoV-infected cells. Red spots indicate upregulated gene transcripts while green spots indicate downregulated gene transcripts. Only genes with a fold change higher than two or lower than minus two (FDR<0.01) were considered.

**Figure 3:**
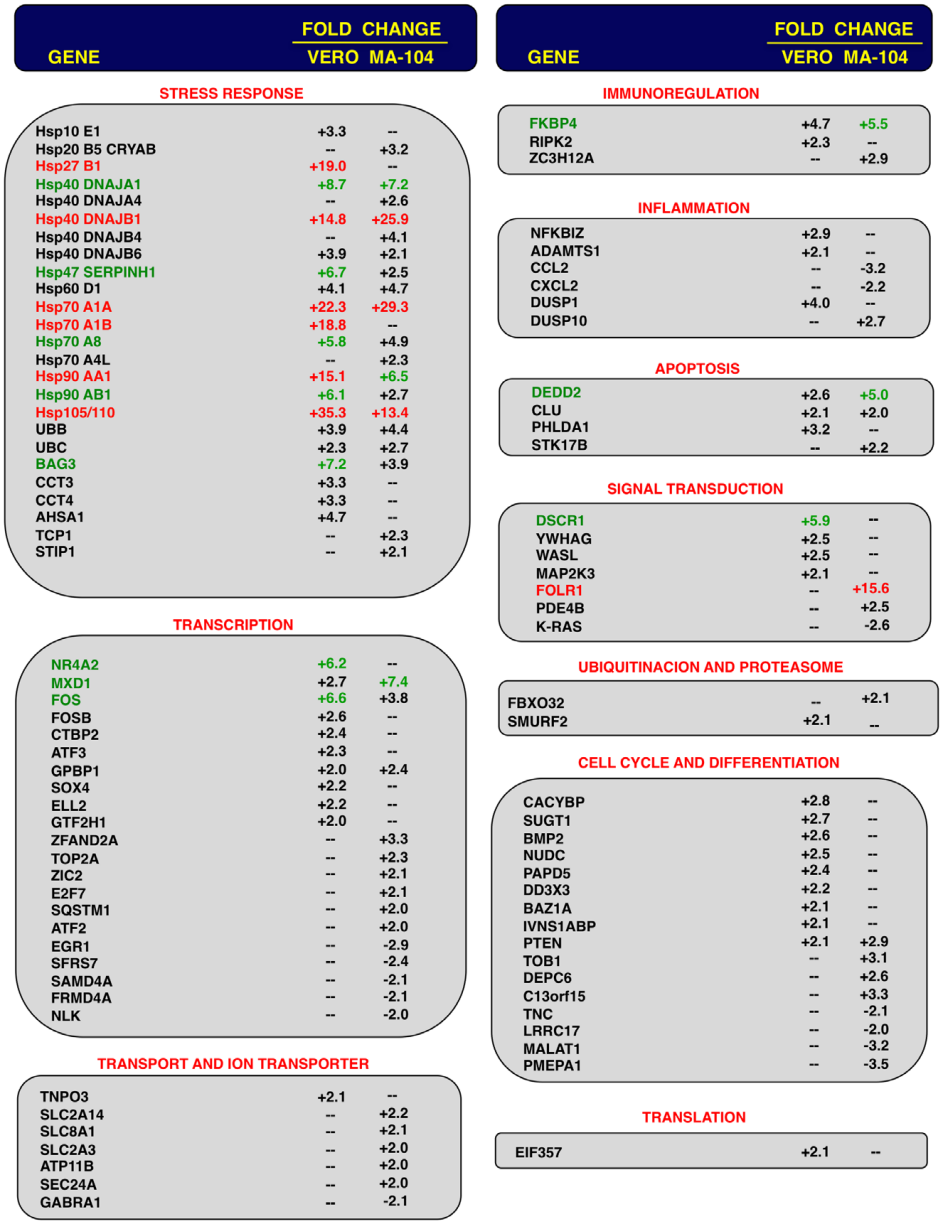
Host cell genes differentially expressed in rSARS-CoV-ΔE versus rSARS-CoV-infected cells using microarrays. Genes differentially expressed in rSARS-CoV-ΔE versus rSARS-CoV-infected Vero E6 and MA-104 cells, were classified according to their main biological functions. Only genes with a fold change higher than two or lower than minus two (FDR<0.01) were considered. –, indicates that the gene is not detected in the array or is not differentially expressed with a fold change higher than two or lower than minus two. Red color indicates genes upregulated more than 10 fold. Green color indicates genes upregulated between 5 and 10 fold, at least in one cell line. For those genes recognized with more than one probe, the value corresponding to the highest upregulation or downregulation is represented.

To analyze the impact of E protein on host gene expression during SARS-CoV infection, the transcriptomes of rSARS-CoV-ΔE and rSARS-CoV-infected Vero E6 and MA-104 cells were compared. Taking into account the data obtained in Figure 1, early (7 hpi in the case of Vero E6, and 24 hpi in the case of MA-104 cells), and late (15 and 65 hpi, in Vero E6 and MA-104 cells, respectively) times post-infection (pi), were analyzed. Microarray-based studies of global gene responses were performed in triplicate in each case. As there are no commercially available microarrays specific for African green monkey species, and the sequence homology between humans and monkeys is very high [59], human U133 plus 2.0 microarrays were used. The results of the microarray analysis have been deposited in the Gene Expression Omnibus (GEO, NCBI, accession code GSE30589). Only those genes showing significant expression changes (i.e., 2.0-fold and false discovery rate (FDR)<0.01) at each time point were selected for further investigation. Comparison of gene expression in cells infected with rSARS-CoV with or without E gene versus mock-infected cells showed that more that 800 cellular genes were differentially expressed at late time post-infection (Fig. 2) and that the number of genes differentially expressed increased over time (i.e. in the case of Vero E6 cells, 4940 annotated genes for rSARS-CoV versus mock-infected cells at 15 hpi, compared to 1324 annotated genes at 7 hpi; for MA-104 cells, 971 annotated genes for rSARS-CoV versus mock-infected cells at 65 hpi, compared to 11 annotated genes at 24 hpi). Interestingly, the number of annotated genes differentially expressed in cells infected with rSARS-CoV-ΔE compared to rSARS-CoV, in which the only difference is the expression of E gene, was reduced to 57 (Vero E6 cells) or to 72 (MA-104 cells) at 15 or 65 hpi, respectively (Fig. 2). These genes were classified according to their most commonly accepted functions (Fig. 3). A high number of genes related to stress responses (19 out of 57 in Vero E6 cells, and 19 out of 72 in MA-104 cells) were differentially expressed, with 2- to 35-fold increases. The pattern of genes upregulated in rSARS-CoV-ΔE compared to rSARS-CoV-infected cells was very similar in Vero E6 and MA-104 cells, and included different isoforms of heat shock protein (hsp) (hsps-10, -27, -40, -60, -70, -90 and -105/110), and different genes encoding ubiquitins and chaperonins (Fig. 3). These data clearly indicated that the cellular stress induced by the infection was significantly reduced in the presence of E protein. Nevertheless, it is worthy to mention that not all cellular stress genes were differentially expressed in cells infected with SARS-CoV lacking E protein versus those infected with rSARS-CoV. In fact, a set of genes coding for different isoforms of hsp40, hsp70, and hsp 90, also modified their expression between −11.0 and and +4.0-fold but to a similar extent in rSARS-CoV-ΔE and rSARS-CoV-infected cells when compared with mock infected ones (Fig. S1).

Differentially expressed genes were also involved in signal transduction, transcription, cell metabolism, immunoregulation, inflammation, apoptosis and cell cycle and differentiation, although to a lower extent (Fig. 3). Among the genes involved in signal transduction, the upregulation of DUSP1 and DUSP10 may be relevant in rSARS-CoV-ΔE attenuation, as these genes are involved in down regulating cellular responses associated with different types of stress. Furthermore, these genes reduce the inflammatory response induced by viral infections by negatively regulating mitogen-activated protein kinase (MAPK) signaling [60]. Accordingly, the expression of the proinflammatory cytokines CCL2 and CXCL2 was reduced in rSARS-CoV-ΔE-infected, compared to rSARS-CoV-infected MA-104 cells.

**Figure 4:**
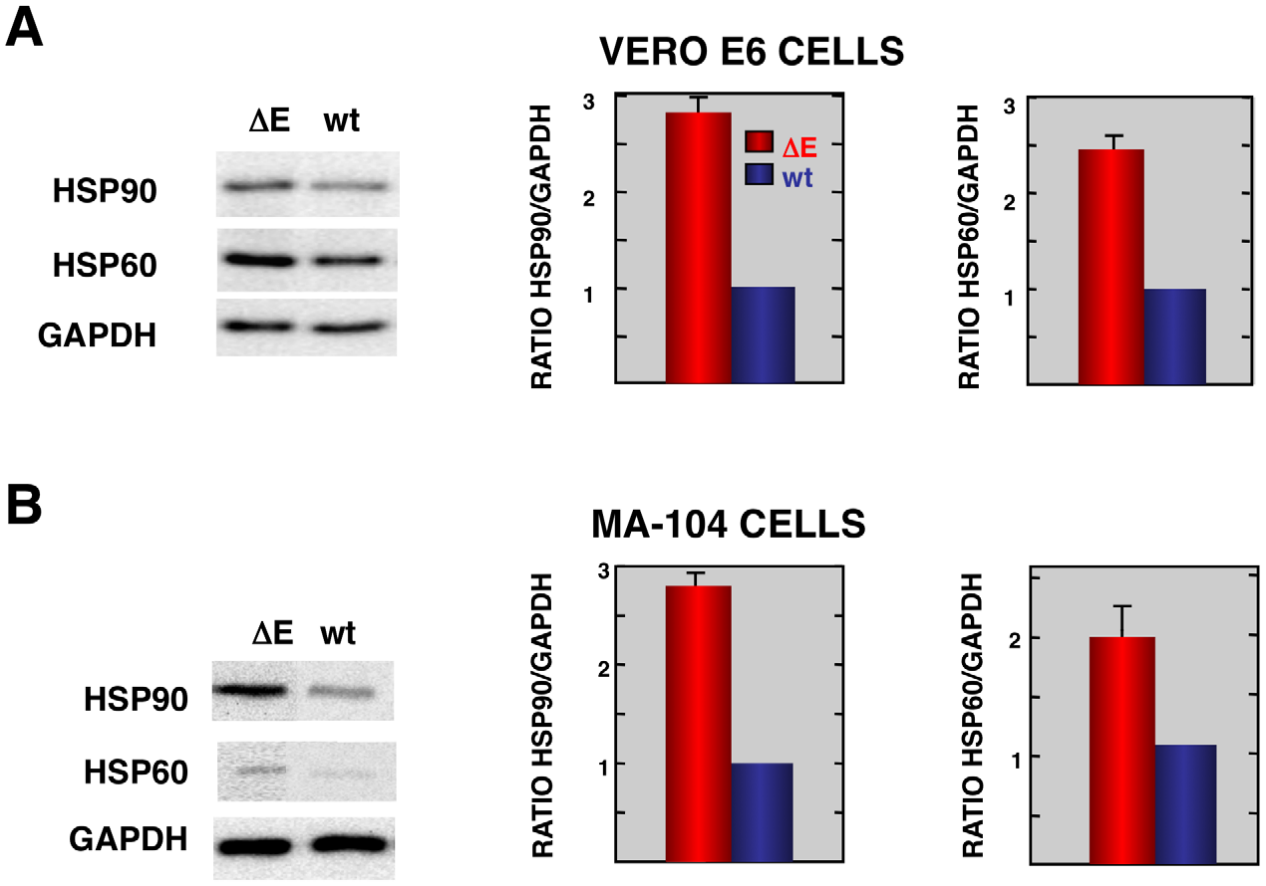
Stress response proteins are differentially expressed in rSARS-CoV-ΔE versus rSARS-CoV-infected cells. Total protein from Vero E6 (A) and MA104 (B) cells infected with rSARS-CoV-ΔE or SARS-CoV was extracted at 22 and 75 hpi, respectively. Levels of hsp90 and hsp60 were normalized to those of GAPDH after Western-blot assay (left panels) and densitometric analysis of the bands (right panels). Columns represent hsp90/GAPDH and hsp60/GAPDH ratios in SARS-CoV-ΔE (red) or SARS-CoV (blue) infected cells. Error bars indicate the standard deviation from three independent experiments.

Consistent with the mRNA results, we detected increases in the levels of representative stress proteins, such as hsp60 and hsp90 although differences were not as great as observed when mRNA levels were assessed (Fig. 4). Lesser effects on protein levels may reflect inhibitory effects of SARS-CoV on non-viral protein synthesis [61] or, alternatively to the presence of pre-existing stress proteins in cells prior to infection.

**Figure 5:**
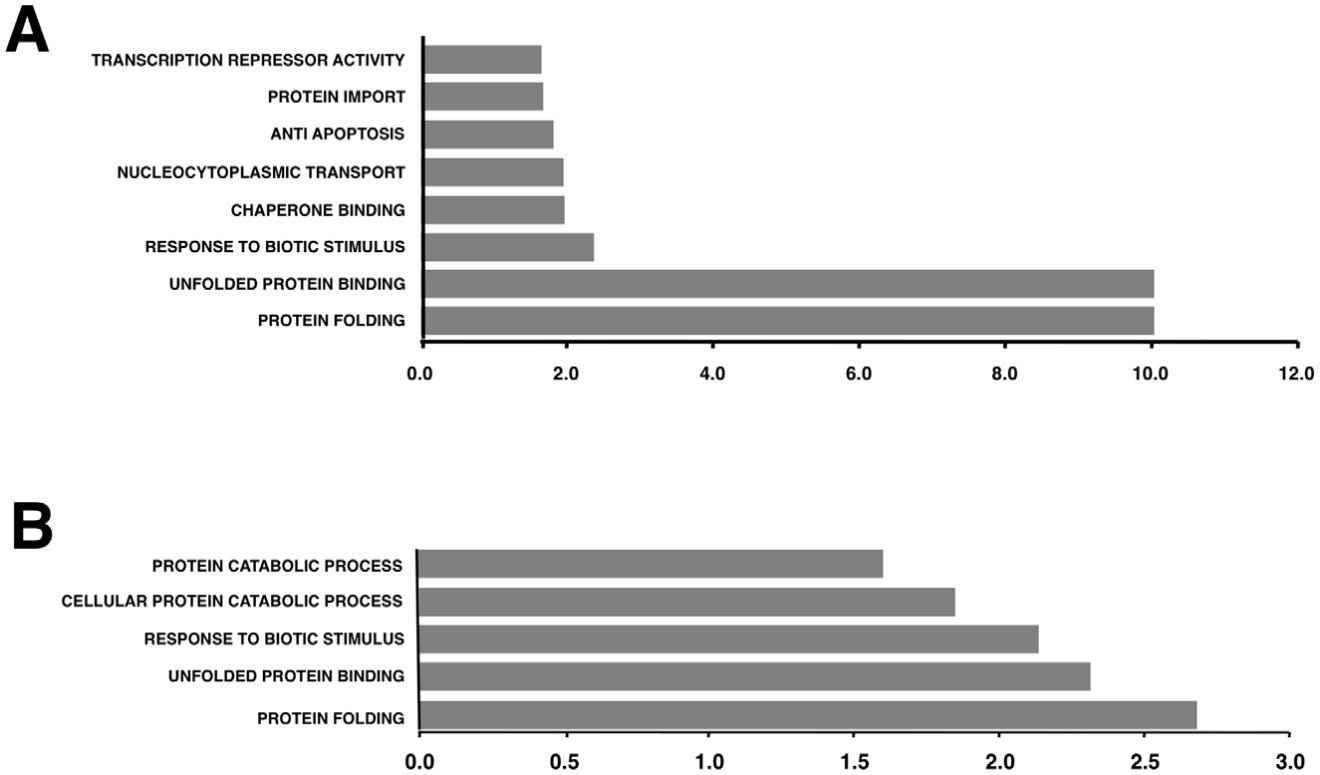
Upregulation of functionally associated genes in rSARS-CoV-ΔE compared to rSARS-CoV-infected cells using microarrays. Gene Sets, based on Gene Ontology terms, that correlate with upregulated genes in Vero E6 cells at 15 hpi (A) and in MA-104 cells at 65 hpi (B). X values: −log10 (FDR-q val).

To better understand the biological relevance of the SARS-CoV E protein on host gene expression, all of the genes that were significantly upregulated or downregulated in rSARS-CoV-ΔE-infected compared to rSARS-CoV-infected Vero E6 and MA-104 cells were clustered in functional groups based on gene ontology (GO) classification. A summary is shown in Fig. 5. In contrast, no enriched GO terms were found for genes that were downregulated in MA-104-infected cells. All of the functionally enriched GO terms were related to cellular stress (chaperone binding, response to biotic stimulus, unfolded protein binding, protein folding), cellular death (anti-apoptosis), cellular transport (protein import, nucleocytoplasmic transport), transcription (transcription repressor activity) and metabolism (protein catabolic process, cellular protein catabolic process). Remarkably, similar, highly significant (FDR<0.01) changes in levels of genes related to cellular stress response to biotic stimulus, unfolded protein binding and protein folding were identified in both Vero E6 and MA-104-infected cells (Fig. 5).

**Figure 6:**
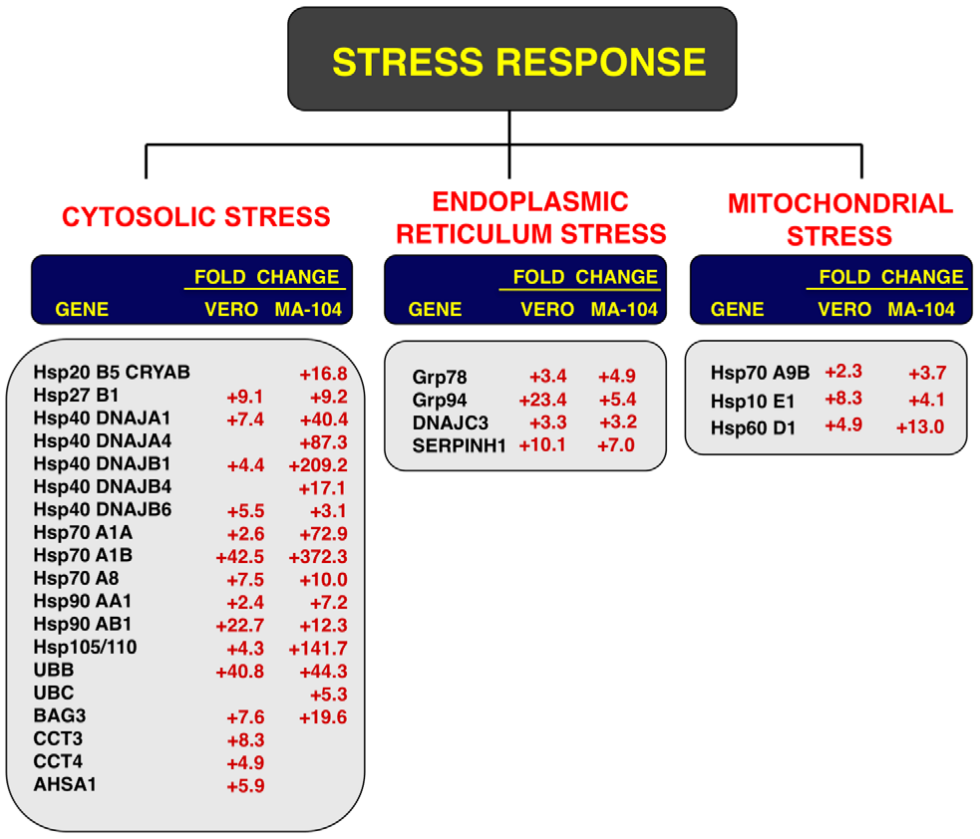
Cellular stress responses induced by rSARS-CoV-ΔE infection. Vero E6 and MA-104 cells were infected with rSARS-CoV-ΔE or SARS-CoV at an moi of 2. Cellular RNAs were extracted at 22 (Vero E6) and 65 (MA-104) hpi and the expression of cellular mRNAs corresponding to cytosolic, ER and mitochondrial stress genes was measured by qRT-PCR. In the case of cytosolic stress, only genes with fold increases >2.5 measured by microarrays were further evaluated by qRT-PCR. Numbers indicate the levels of gene expression in rSARS-CoV-ΔE compared to SARS-CoV-infected cells. Three independent experiments were analyzed with similar results in all cases.

To validate the results obtained with the cDNA microarrays, the differential expression of a wide set of cellular genes observed in cells infected with rSARS-CoV with or without the E gene was evaluated by qRT-PCR. 18S ribosomal RNA (rRNA) was used in all cases to normalize the data because differences in levels of this RNA were always lower than 1.5-fold and because the 18S rRNA has also been used successfully in similar reports [62], [63]. The patterns of differential gene expression obtained by qRT-PCR analysis were similar to those observed with the microarray data (Figs. 3 and 6), validating the results obtained with both techniques. Nevertheless, in the case of genes with large differences in expression between rSARS-CoV and rSARS-CoV-ΔE-infected cells determined using microarrays, changes were even larger when evaluated by qRT-PCR.

### Influence of SARS-CoV E protein on cell stress response

To confirm the effect of the E protein on the stress response, total RNA from infected cell cultures were analyzed at different times pi (15, 22 and 28 hpi in the case of Vero E6 cells, and 24, 48, 65 and 75 hpi, in the case of MA-104 cells) for the expression of genes related to cytosolic (hsp70 A1B and hsp90 AB1), ER (hspA5/GRP78), and mitochondrial (hsp60 D1) stress by qRT-PCR. Maximal differences in the upregulation of the three types of stress responses in rSARS-CoV-ΔE compared to rSARS-CoV were observed at 22 and 65 hpi in Vero E6 and MA-104 cells, respectively (Fig. S2). Consequently, these time points were selected to further analyze the stress responses elicited by these viruses (Fig. 6). Using microarrays, we observed that nineteen genes involved in cytosolic stress were upregulated at least 2.5-fold (FDR<0.01) in rSARS-CoV-ΔE-infected compared to rSARS-CoV-infected Vero E6 cells (15 hpi) and MA-104 cells (65 hpi). Changes in expression of these cytosolic stress genes were confirmed by qRT-PCR (Fig. 6) and shown to be highly significant (from 2.4 to 42.5-fold in Vero E6 cells, and from 3.1 to 372.3-fold in MA-104 cells). In addition, we confirmed the effect of E protein on ER (GRP78, GRP94, DNAJC3 and SERPINH1) and mitochondrial (hspA9, hsp10 E1, and hsp60 D1) stress, using infected Vero E6 and MA-104 cells (Fig. 6), with differences in gene expression that were up to 23.4 or 13.0-fold greater for ER and mitochondrial stress, respectively. These data reinforced the conclusion that SARS-CoV E protein reduced cellular stress induced by SARS-CoV, and that this reduction affected the cytosol, ER, and mitochondria.

In the experiments described above, virus without E protein was passaged 16 times, resulting in a virus with a 10-fold increase in titer, and a single point mutation in the S gene. To rule out the possibility that the mutation in the S gene was responsible for the observed increase in cellular stress, and not the absence of E protein, the induction of stress genes in cells infected with rSARS-CoV-ΔE-p1, which has an RNA genome sequence identical to that of the parental virus except for the deletion of gene E, was analyzed. Total RNA from Vero E6 cultures infected with the original viruses (P1) with or without E protein, and with the virus lacking E protein passaged 16 times, was extracted at 22 hpi. The expression of cellular genes involved in cytosolic, ER, and mitochondrial stress was evaluated by qRT-PCR. Cellular stress genes were upregulated to similar extents in cells infected with the viruses lacking the E gene (either from P1 or P16) compared to cells infected with virus expressing the E gene (Fig. S3). These data indicated that the mutation in gene S was not responsible for the observed differences in stress response, and confirmed that E protein itself down regulated the cellular stress in virus-infected cells.

**Figure 7:**
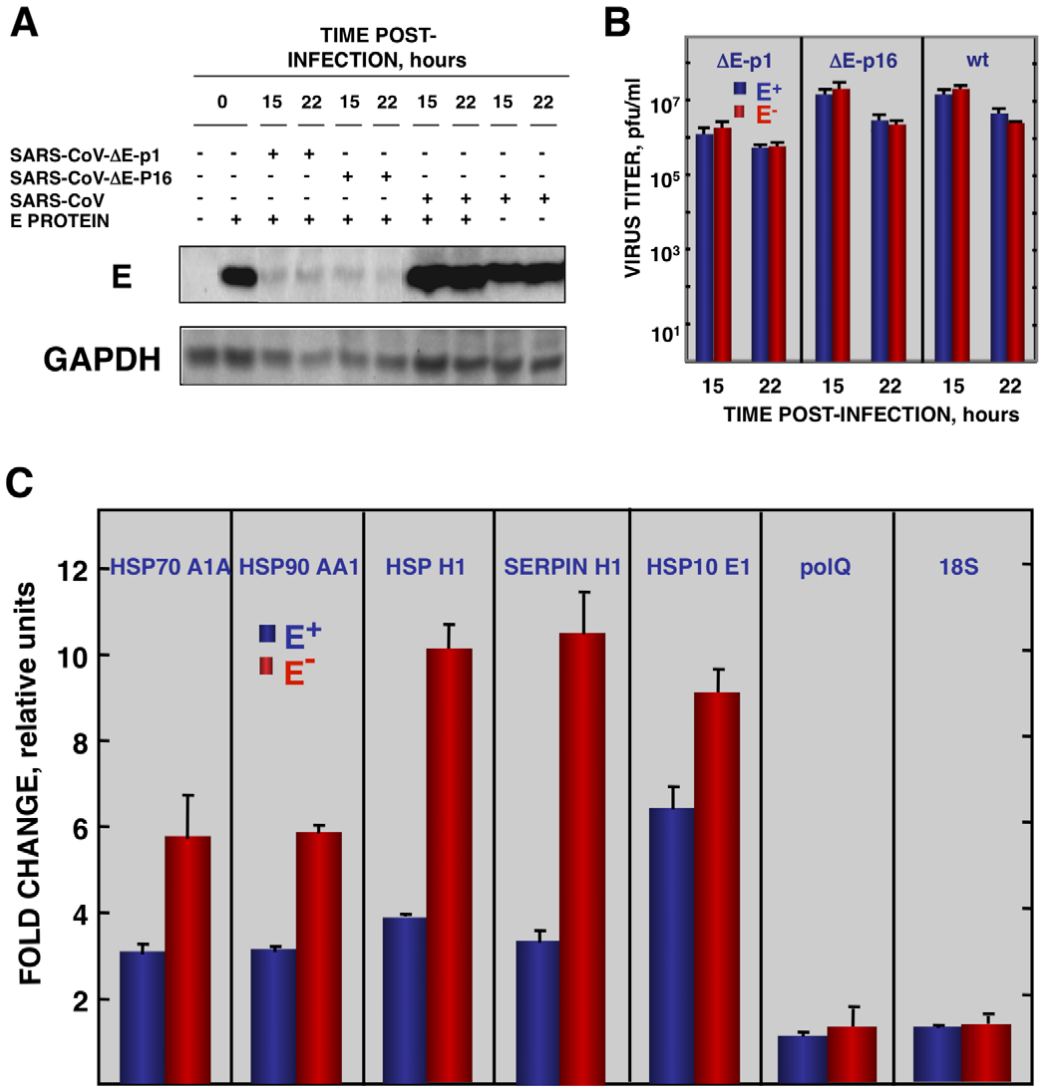
Effect of SARS-CoV E protein on stress induced by SARS-CoV infection. Vero E6 cells infected at an moi of 0.5 with rSARS-CoV-ΔE-P1 or rSARS-CoV were transfected with a plasmid expressing E protein (E^+^) or with the empty plasmid (E^−^) as control. (A) Accumulation of SARS-CoV E protein and GAPDH as a loading control, at 15 and 22 hpi were evaluated by Western blot. (B) Virus titers in the presence or absence of E protein provided in trans were evaluated at 15 and 22 hpi. (C) At 22 hpi, cellular RNAs were extracted, and the expression of the stress-induced genes hspA1A, hsp90AA1, hspH1, SERPINH1, and hspE1, and that of polQ and 18S rRNA, as controls, were analyzed by qRT-PCR. In each case, the expression levels of mRNAs encoding representative cell stress proteins were evaluated in rSARS-CoV-ΔE-P1-infected cells in relation to rSARS-CoV-infected cells. Bars represent standard deviations of the mean from three experiments.

To reinforce the conclusion that SARS-CoV E protein was responsible for the reduction of cellular stress, we transfected the E gene into rSARS-CoV-ΔE-infected cells together with controls. Vero E6 cells were infected with viruses lacking the E gene (P1 and P16) or with virus expressing the E gene, and 90 min later, cells were transfected with the plasmid pcDNA3.1-E, encoding the E protein, or with empty plasmid as a control. E protein was expressed in cells transfected with plasmids expressing this protein, although levels were 10-fold lower than in SARS-CoV infected cells, as shown by Western-blot analysis (Fig. 7A). As an additional control, the effect of E protein expression on the replication of SARS-CoV with or without E gene was studied (Fig. 7B). E protein added in trans had no significant effect on rSARS-CoV-P16 or rSARS-CoV-ΔE titers, indicating that the absence of E protein in rSARS-CoV-ΔE, and not the amount of virus produced, was responsible for the increase in cell stress response. The expression of stress genes hsp70 A1A, hsp90 AA1, hspH1, SERPINH1, and hsp10 E1 in cells infected with rSARS-CoV-ΔE viruses (P1 and P16) or rSARS-CoV, in the presence or absence of the transfected E gene, was analyzed by qRT-PCR (Figs. 7C and S4). The expression of all analyzed stress-induced genes was clearly upregulated in cells infected with virus lacking E protein, compared to those infected with rSARS-CoV. When E protein was provided in trans, the expression of these genes in rSARS-CoV-ΔE-infected cells was clearly reduced. To analyze whether the decreased expression of stress-related genes in the presence of E protein was specific, the expression of the gene encoding DNA polymerase theta (polQ) was evaluated. No significant differences were observed in the expression of polQ, irrespective of the presence or absence of E protein (Figs. 7C and S4), suggesting that the reduction of stress-related genes was specific. In addition, the expression of 18S rRNA was analyzed as an endogenous control for the amount of RNA in all samples (Figs. 7C and S4). These data indicated that E protein reduced the stress caused by SARS-CoV infection.

**Figure 8:**
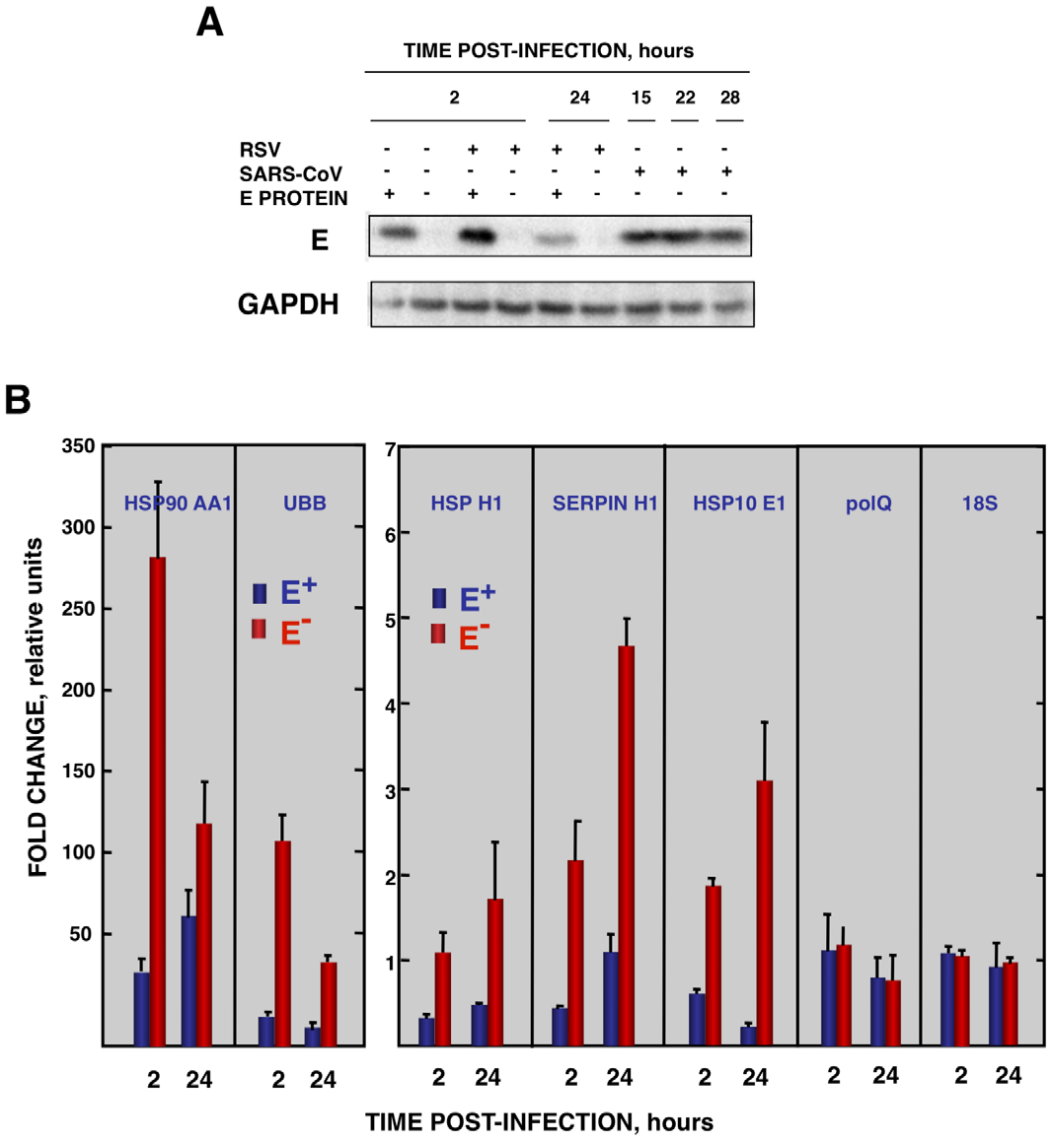
Effect of SARS-CoV E protein on the stress induced by RSV infection. Vero E6 cells transfected with a plasmid expressing SARS-CoV E protein (E^+^) or with the empty plasmid (E^−^) were infected with RSV at a moi 2. (A) Accumulation of SARS-CoV E protein and GAPDH as a loading control, at 2 and 24 hpi in the case of RSV-infected cells and at 15, 22, and 28 hpi in the case of SARS-CoV-infected cells, was evaluated by Western blot. (B) Intracellular RNA was extracted at 2 and 24 hours post-RSV infection and the expression of cellular stress genes, polQ and 18S rRNA, was measured by qRT-PCR. In each case, levels of expression in infected cells were compared to those in mock-infected ones. Bars represent standard deviations of the mean of results from three experiments.

To analyze whether E protein alone could reduce the cellular stress caused by another virus, the effect of SARS-CoV E protein on the stress induced by RSV was analyzed. Vero E6 cells were transfected with a plasmid encoding E protein or with empty plasmid as control. At 24 hours post-transfection (hpt), Vero E6 cells were infected with RSV or left uninfected, and RNA was extracted at the indicated hpi. The expression of E protein in cells infected with RSV was confirmed by Western-blot analysis and the levels were similar to those of rSARS-CoV infected cells (Fig. 8A). In addition, no significant effect of E protein expression on RSV titers was detected. The expression of the stress response genes hsp90 AA1, UBB, hspH1, SERPINH1 and hsp10 E1 was analyzed in the presence or absence of SARS-CoV E protein by qRT-PCR (Fig. 8B). The expression of these stress response genes was significantly induced by RSV infection at almost all times (Fig. 8B). In the presence of E protein, the induction of these stress genes was significantly reduced (Fig. 8B) in a specific manner as no significant differences were observed in the expression of polQ gene, irrespective of the presence or absence of E protein (Fig. 8B). These data indicated that SARS-CoV E protein alone reduced different types of stress, such as cytosolic (genes hsp90 AA1, UBB, hspH1), ER (gene SERPINH1) and mitochondrial stress (gene hsp10 E1), produced by infection with at least two different respiratory viruses (SARS-CoV and RSV).

**Figure 9:**
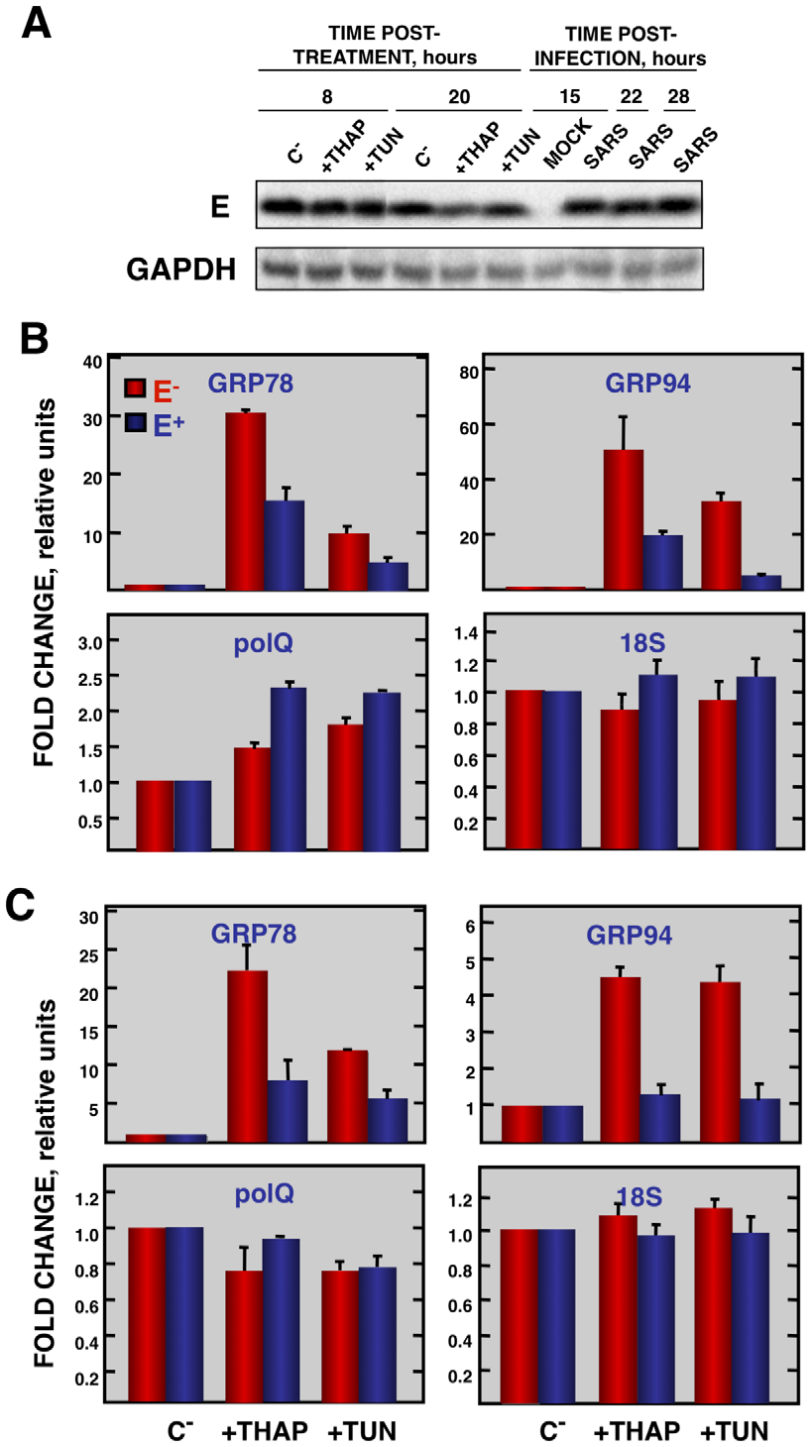
Effect of SARS-CoV E protein on the induction of ER stress caused by drugs. Vero E6 and MA-104 cells were transfected with a plasmid expressing SARS-CoV E protein (E^+^) or with the empty plasmid (E^−^). At 24 hpt, the cells were treated with 1000 nM thapsigargin (+thap), 2 µg/ml of tunicamycin (+tun) or left untreated (−). (A) Levels of SARS-CoV E and GAPDH (loading control) at 8 and 20 h post treatment in Vero E6 cells were determined by Western blot. The expression of the stress induced genes, GRP78 and GRP94 ER and that of polQ and 18S rRNA was evaluated by qRT-PCR in Vero E6 (B) or MA-104 (C) cells. In each case, levels of expression in treated cells were compared to non-treated cells. Bars represent the standard deviations from the mean in three independent experiments.

Coronavirus infection induces ER stress [64] due to the extensive use of intracellular membranes for the generation of replication complexes and for the assembly of virus particles [36], [65]. In addition, viral glycoproteins can induce ER stress during infection as a result of incomplete glycosylation and incorrect folding or accumulation in the ER lumen [66], [67]. Accordingly, we decided to focus our attention on ER stress. To determine whether E protein alone was responsible for the downregulation of the ER stress response, Vero E6 and MA-104 cells were transfected with a plasmid encoding SARS-CoV E protein or with empty plasmid as a control. At 24 hpt cell cultures were treated with thapsigargin and tunicamycin, which induce ER stress by altering intracellular Ca^++^ levels or by preventing protein glycosylation, respectively [68], for 8 or 20 h, or left untreated. The levels of E protein were monitored by Western-blot analysis and were similar to E protein levels after SARS-CoV infection of Vero E6 (Fig. 9A) or MA-104 cell (data not shown). Total cellular RNAs were collected and the expression of the ER-stress inducible genes GRP78 and GRP94 was evaluated by qRT-PCR. The effect of E protein expression at the times post-induction when upregulation of these genes was highest is shown (Fig. 9B and C). Treatment with thapsigargin and tunicamycin clearly induced the expression of ER stress genes in Vero E6 and MA-104 cells transfected with the empty plasmid (Fig. 9B and C). The expression of GRP78 and GRP94 was significantly reduced in the presence of E protein (Fig. 9B and C). No decrease in the expression of polQ gene was observed in the presence of E gene, suggesting that the reduction in the expression of stress related genes was specific (Fig. 9B and C). These data indicated that E protein alone was sufficient to reduce cellular stress caused by different mechanisms.

### Modulation of UPR by SARS-CoV E protein

Cells induce the UPR to reduce the burden imposed by unfolded or misfolded proteins in the ER. To analyze the mechanisms by which the E protein can reduce ER stress, the effect of E protein on the three branches of the UPR (PERK, ATF6, and IRE-1) was analyzed. The PERK pathway involves the phosphorylation and subsequent activation of this kinase. Accordingly, the levels of phosphorylated PERK in Vero E6 cells infected with rSARS-CoV-ΔE or rSARS-CoV were compared at different times pi. As a control, levels of the house-keeping gene GAPDH were measured and used for normalization. Phosphorylated PERK was detectable in rSARS-CoV-ΔE and wt-infected cells at 6 hpi, in contrast to mock-infected cells, in which no phosphorylated PERK was detected. No significant differences in the phosphorylation levels of PERK were detected between cells infected with rSARS-CoV with or without E protein (Fig. S5), suggesting that E protein had no significant influence on the phosphorylation of PERK.

To analyze whether E protein inhibited the ATF6 pathway, the extent of ATF6α processing in cells infected with rSARS-CoV with or without the E gene, or mock-infected cells was measured by Western blot using an ATF6-specific antibody that recognizes the full-length and the cleaved N-terminal domain of the protein. No significant activation of ATF6 was observed in infected cells, compared to mock-infected cells (data not shown), suggesting that SARS-CoV infection did not efficiently activate this pathway.

**Figure 10:**
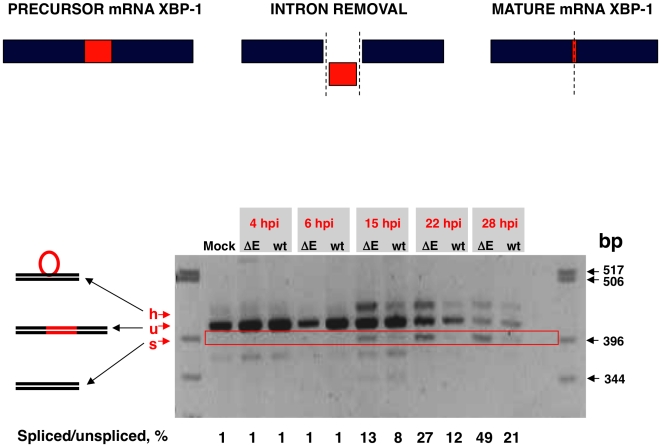
Activation of the IRE-1 pathway in rSARS-CoV-ΔE-infected cells. Vero E6 cells were infected with rSARS-CoV-ΔE or rSARS-CoV at an moi of 2. Splicing of XBP-1 mRNA was analyzed at different times pi using oligonucleotides flanking the splicing region. Numbers below the gel represent the percentage of spliced/unspliced forms of XBP-1.

Activation of IRE-1 mediates cytoplasmic splicing of the mRNA encoding the transcription factor XBP-1, leading to a frame shift and subsequent translation of a functional XBP-1 transcription factor. To evaluate whether SARS-CoV E protein has an impact on the activation of this pathway, Vero E6 cells were infected with rSARS-CoV with or without the E gene and RNA was collected at different times pi. RT-PCR was used to amplify fragments representing both the unspliced (u) and spliced (s) forms of XBP-1 mRNA, differing by 26 nt [69] (Fig. 10). The relative abundance of these XBP-1 mRNAs was independent of PCR efficiency as the corresponding mRNAs were amplified using the same primer pair. A third slowly migrating species (h), corresponding to a heterohybrid formed by the amplified unspliced and spliced forms was also detected (Fig. 10). The activation of IRE-1 was estimated as a ratio between the spliced and unspliced forms of XBP-1. Levels of spliced XBP-1 were higher in rSARS-CoV-ΔE-infected compared to rSARS-CoV infected cells from 15 to 28 hpi (Fig. 10). This result indicated that in the presence of E protein, activation of the XBP-1 pathway was reduced.

### Inhibition of apoptotic cell death by SARS-CoV E protein

**Figure 11:**
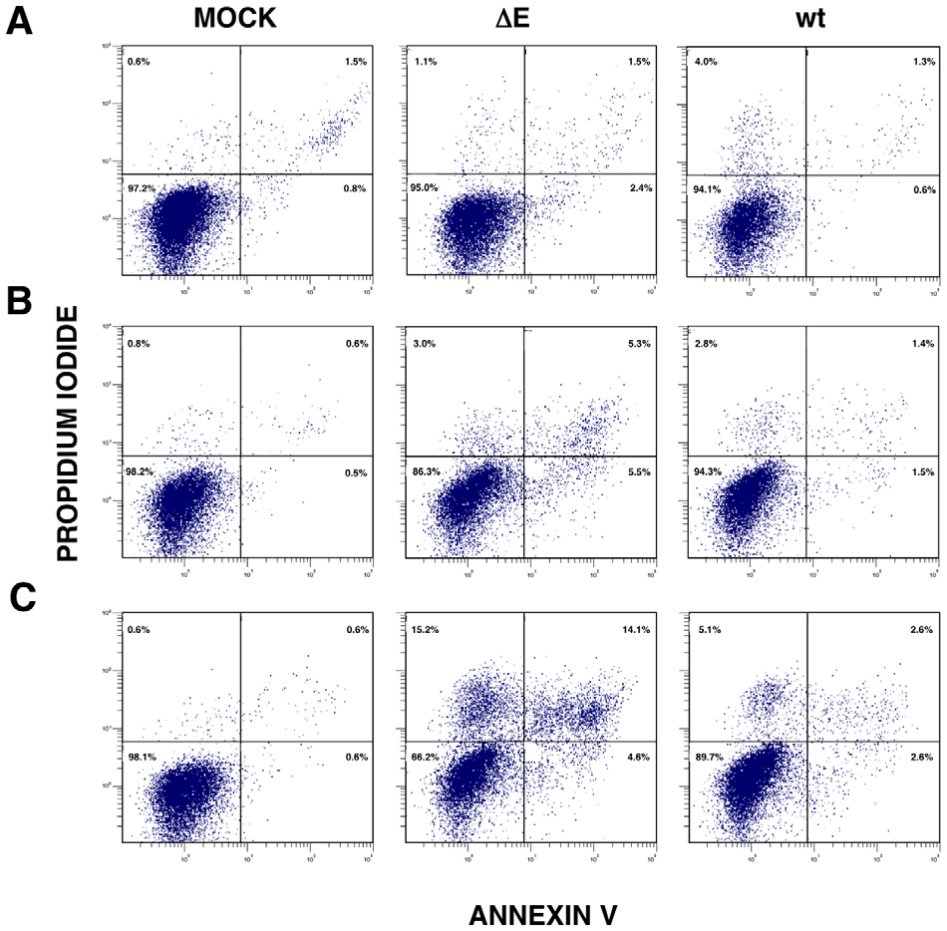
rSARS-CoV-ΔE-induced apoptosis. Apoptosis levels in mock, rSARS-CoV-ΔE and rSARS-CoV-infected cells were evaluated at 4 (A), 15 (B) and 24 (C) hpi by flow cytometry. Annexin V-PI double staining was performed to differentiate cells in early apoptosis (Annexin V^+^, PI^−^) from those in late apoptosis (Annexin V^+^, PI^+^).

Persistent or intense ER stress can trigger apoptosis [41]. To analyze whether SARS-CoV E protein modulated apoptosis induced by SARS-CoV infection, the induction of apoptosis was analyzed in cells infected with rSARS-CoV lacking or expressing E gene. Cells infected either with rSARS-CoV or rSARS-CoV-ΔE were simultaneously stained with propidium iodide (PI) and Annexin V, and monitored by flow cytometry. Mock infected cells remained viable (Annexin V^−^, PI^−^) throughout the experiment, indicating that the treatment did not induce apoptosis by itself (Fig. 11). rSARS-CoV induced low levels of apoptosis (Annexin V^+^) from 15 hpi, and a minor cell population in late apoptosis (Annexin V^+^, PI^+^) was evident from 24 hpi (Fig. 11). rSARS-CoV-ΔE triggered apoptosis more rapidly and to a greater extent than rSARS-CoV, with a 3 to 4-fold increase in early apoptotic cells at 4 and 15 hpi, and a 4 and 5- fold increase in late apoptotic cells between 15 and 24 hpi (Fig. 11).

## Discussion

We previously showed that rSARS-CoV-ΔE is attenuated *in vivo* [26], [27]. In this work, to identify possible mechanisms for this attenuation, the effect of E protein on host cell responses during virus infection was analyzed by comparing the transcriptome of rSARS-CoV-ΔE and rSARS-CoV-infected cells. Among the genes differentially expressed, a large number of genes corresponding to cellular stress were upregulated in rSARS-CoV-ΔE compared to wt virus infected cells, clearly indicating that the presence SARS-CoV E protein reduced the stress response during infection. Upregulation of the stress response was also confirmed at the protein level, as the expression of representative stress response proteins, such as hsp60 and hsp90 was also increased. The addition of E protein in trans reversed the increase in stress response gene expression observed in rSARS-CoV-ΔE-infected cells, confirming the specific suppression of the stress response by E protein. Interestingly, levels of E protein were 10-fold lower than those expressed in SARS-CoV-infected cells, but were sufficient to reduce the increase in stress response genes, indicating the robust effect of E protein. In addition, rSARS-CoV-ΔE titers were not significantly increased by providing E protein in trans, probably due to the low levels of E protein expressed in rSARS-CoV-ΔE infected cells, indicating that the presence or absence of E protein, and not the amount of virus, was responsible for the increase in stress response and apoptosis. In addition, stress induced by another virus, RSV, was also downregulated by SARS-CoV E protein. Furthermore, expression of E protein in the absence of virus infection reduced stress induced by tunicamycin or thapsigargin. SARS-CoV E protein also inhibited a subset of the stress response. Specifically, E protein inhibited the activation of the XBP-1-mediated pathway of the UPR, and apoptosis induced by SARS-CoV. We have shown that in MA-104 cells infected with rSARS-CoV-ΔE, two important pro-inflammatory cytokines (CCL2/MCP-1 and CXCL2/macrophage inflammatory protein 2 [MIP-2]) were downregulated, indicating that the E protein reduces virus-induced inflammation.

SARS-CoV is the most pathogenic human coronavirus known [70]. Besides pneumonia, SARS-CoV causes diarrhea [71], lymphopenia [72], haematological disorders [47], pulmonary vasculitis, and thrombosis [73], [74]. In previous reports, we showed that rSARS-CoV-ΔE was attenuated in hamsters and hACE2 transgenic mice [26], [27]. The relevance of virus-host interaction in virus attenuation is high as differences in virulence are frequently due to differences in host responses, rather than to virus growth kinetics [75], [76].

Coronavirus infection induces an ER stress response due to the extensive use of ER membranes for RNA synthesis [35], [36] and virion assembly at the ER-Golgi intermediate compartment [64], [77]. Further, it has been shown that SARS-CoV structural proteins S, 6, and 3a [66], [78], [79], [80], and the accessory protein 8ab [81] induce ER stress responses. Using genomic approaches, the upregulation of stress genes in SARS-CoV-infected Huh-7 [82], Vero [59], and blood mononuclear cells [83], [84] has been reported in cell cultures and also *in vivo* [75], [85]. We show, for the first time, that SARS-CoV E protein limits the stress response elicited by SARS-CoV infection, which probably represents a selective advantage for the virus. In fact, we have shown that rSARS-CoV-ΔE is cleared faster than rSARS-CoV with E protein [26], [27]. We observed that genes related to hsps were upregulated in rSARS-CoV-ΔE infected compared to wt virus-infected cells. The presence of hsps on the cell surface facilitates the elimination of infected cells by natural killer (NK) and T cell subsets [32]. Hsps facilitate the presentation of antigenic peptides by the major histocompatibility complex I (MHC I), helping clearance of infected cells by CD8^+^ T cells [86].

SARS-CoV E protein expressed in trans reduced the stress response induced by rSARS-CoV-ΔE, by a heterologous virus such as RSV (without affecting the amount of virus in both cases), and by non-viral agents, such as thapsigargin and tunicamycin. Therefore, E protein limited the ER stress caused by the unbalance of ER Ca^++^ ion concentrations, and by the inhibition of N-glycosylation leading to the accumulation of misfolded or unfolded proteins. Overall, these results showed that the downregulation of the stress response by SARS-CoV E protein was a general phenomenon.

In order to analyze the specific pathways modulated by SARS-CoV E protein, the three branches of the UPR were analyzed. Only the XBP-1 pathway was significantly activated in cells infected with rSARS-CoV-ΔE compared to infection with the wt virus. Possibly, the partial activation of the UPR was not sufficient to alleviate cellular stress, and cell apoptosis was induced to help virus clearance [31], [41]. The ectopic expression of coronavirus E protein induces apoptosis in the absence of infection [87], [88], whereas in this manuscript we describe that the expression of E protein in the context of SARS-CoV infection, limited the levels of apoptosis in infected cells, which may represent an advantage for virus production and dissemination [89]. This is not surprising, as previous experiments were performed in transfected cells and not in the context of viral infection, and as many other viral proteins such as 3C-like protease, spike, membrane, nucleocapsid, 3a, 3b, and 7a (reviewed by Tan et al. in [45]), and proteins 6, 7b, and 8a [78], [90], [91] also elicit apoptosis. Removal of the E gene from SARS-CoV led to an increase in stress responses and UPR. Nevertheless, the stress and UPR responses were not able to balance the homeostasis of the system and apoptosis was increased as a defense mechanism that may have contributed to the attenuation observed in rSARS-CoV-ΔE-infected hamsters and mice [26], [27]. Overall, these data indicate that the regulatory influence of E protein on signaling pathways leading to apoptosis still needs further clarification. The control of the stress response and apoptosis by a viral protein has also been observed in infections by human cytomegalovirus, in which the UL38 protein suppresses ER stress-induced death, preventing premature cell death and facilitating efficient virus replication [92], [93].

The expression of genes leading to exuberant inflammation has been associated with SARS-CoV-induced pathology [75], [76]. The upregulation of stress genes observed in SARS-CoV-infected cells when the E gene was deleted probably diminished proinflammatory processes, leading to a decrease in pathology [94], [95]. In fact, we have observed that MAPK phosphatases DUSP1 and DUSP10 were upregulated in rSARS-CoV-ΔE-infected cells when compared to wt virus-infected cells. DUSP proteins are critical regulators of innate immune responses [96]. Using DUSP1 and DUSP10 knock out cell cultures and mice, it has been shown that these genes limit the expression of inflammatory genes such as TNF, IL-6, CCL2/MCP-1, CCL3, CCL4 and CXCL2/MIP-2 [60], [97], [98], [99]. Interestingly, we observed a decrease in the expression of CXCL2/MIP-2 and CCL2/MCP-1 in rSARS-CoV-ΔE infected MA-104 cells compared to wt virus-infected cells, probably contributing to the reduction of lung inflammation that we observed *in vivo* [26], [27]. In human SARS, increases in IL-6, CCL2/MCP-1 and CXCL10/IP-10 expression were detected in the lungs of human patients with fatal SARS [48], [49], [100]. Furthermore, persistent expression of CCL2/MCP-1, CXCL9/MIG and CXCL10/IP-10 was observed in the blood of SARS patients with fatal disease [48], [49], [100], reinforcing the idea that elevated expression of proinflammatory cytokines significantly contributes to the pathogenicity of the virus.

In summary, we found that deletion of the E gene from SARS-CoV increased the expression of host genes involved in stress response and immunoregulation, among others, and decreased those involved in inflammation. Further, SARS-CoV E protein reduced the stress caused by two viruses, SARS-CoV and RSV, and by drugs. E protein may represent a novel strategy used by SARS-CoV to increase its virulence and may also serve as a potential therapeutic target in outbreaks of SARS-CoV or other coronaviruses.

## Materials and Methods

### Virus

rSARS-CoV and rSARS-CoV-ΔE were rescued from infectious cDNA clones as previously described [26], [101]. rSARS-CoV-ΔE was passaged 16 times in Vero E6 cells and characterized *in vitro* and *in vivo* (rSARS-CoV-ΔE-P16) [56]. Remarkably, only a single mutation, at position 23312, which resulted in a serine to phenylalanine mutation in the gene S, was detected in the rSARS-CoV-ΔE passaged 16 times [56]. All work with infectious viruses was performed in biosafety level (BSL) 3 facilities by personnel wearing positive-pressure air purifying respirators (3M HEPA AirMate, St. Paul, MN).

### Cells

African Green monkey kidney-derived Vero E6 cells were kindly provided by Eric Snijder (Medical Center, University of Leiden, The Netherlands). African monkey kidney-derived MA-104 cells were kindly provided by J. Buesa (Universidad de Valencia, Valencia, Spain). Human colon carcinoma-derived CaCo-2 cells were obtained from the *European Collection of Cell Cultures* (EACC 86010202). Human hepatocarcinoma-derived Huh7 cells were provided by R. Bartenschlager (Department for Molecular Biology, University of Heidelberg, Germany). Rhesus monkey kidney-derived FRhK-4 cells were obtained from the *American Type Culture Collection* (ATCC CRL-1688). Porcine kidney-derived PK15 cells were provided by A. Carrascosa (Centro de Biología Molecular, Madrid, Spain). Human hepatocarcinoma-derived HepG2 cells were provided by M. Esteban (Centro Nacional de Biotecnología, Madrid, Spain). Human kidney-derived 293 cells were obtained from the *American Type Culture Collection* (ATCC CRL-1573). The 293-derived clone 293T, which expresses the SV40 T antigen, was obtained from the *American Type Culture Collection* (ATCC CRL-11268). In all cases, cells were grown in Dulbecco's modified Eagle's medium (GIBCO) supplemented with 25 mM HEPES and 10% fetal bovine serum (Biowhittaker). Virus titrations were performed in Vero E6 cells following standard procedures using closed flasks or plates sealed in plastic bags, as previously described [26].

### rSARS-CoV growth kinetics

Subconfluent monolayers (90% confluency) of Vero E6 and MA-104 cells were infected at an moi of 2 with rSARS-CoV-ΔE, or rSARS-CoV. Culture supernatants were collected at different hpi and virus titer was determined as previously described [26].

### Indirect immunofluorescence assay

Subconfluent Vero E6, MA-104, CaCo-2, Huh7, FRhK-4, PK15, HepG2, 293 and 293T cells grown in 9 cm^2^ flasks were infected at an moi of 1, 3 or 5. At different times pi, cells were washed in ice-cold phosphate-buffered saline (PBS) and fixed with 4% paraformaldehyde for 30 min at room temperature. The cells were then permeabilized with 0.2% saponin in blocking solution (PBS, pH 7.4, containing 10% FBS) for 1 h at room temperature and incubated with a SARS-CoV N protein-specific monoclonal antibody (SA46-4), kindly provided by Ying Fang (Center for Infectious Disease Research and Vaccinology, Brookings, South Dakota, USA) for 90 min at room temperature. Cells were then washed three times with PBS, incubated with Alexa 488-conjugated mouse antibodies (Molecular Probes) at 1∶500 dilution in blocking solution for 30 min at room temperature and washed five times with PBS. The slides were removed, mounted with glass coverslips and analyzed with a Zeiss Axiophot fluorescence microscope.

### Microarray analysis

Vero E6 or MA-104 cells were mock-infected or infected at an moi of 2 with rSARS-CoV or rSARS-CoV-ΔE. Total RNA was extracted using a RNeasy mini kit (Qiagen) according to the manufacturer's instructions and RNA integrity was measured in a bioanalyzer (Agilent Technologies, Inc.). RNAs were biotin-labeled using the *One cycle target-labeling kit* (Affymetrix, Santa Clara, CA). Briefly, cDNA was synthesized from 5 µg total RNA using an oligo-dT primer with a T7 RNA polymerase promoter site added to the 3′ end. After second-strand synthesis, *in vitro* transcription was performed using T7 RNA polymerase to produce biotin-labeled cRNA. cRNA preparations (15 µg) were fragmented at 94°C for 35 min into 35–200 bases in length and added to a hybridization solution (100 mM 4-morpholinopropanosulfonate acid, 1 M Na^+^, 20 mM EDTA and 0,01% Tween-20). The cRNAs (10 µg) were hybridized to Human Genome U133 plus 2.0 Arrays (Affymetrix, Santa Clara, CA) at 45°C for 16 hours. The arrays were stained with streptavidin-phycoerythrin and read at 1.56 µm in a GeneChip Scanner 3000 7G System (Affymetrix, Santa Clara, CA). Three independent microarrays were hybridized for each experiment.

### Microarray data analysis

Data analysis was performed with the system affylma GUI R [102]. Robust Multi-array Analysis (RMA) algorithm was used for background correction, normalization and presentation of the expression levels [103]. Next, analysis of differential expression was performed with the Bayes t-statistics using microarray data (limma) linear models, included in the affylmGUI package. P-values were corrected for multiple-testing using the Benjamini-Hochberg's method (False Discovery Rate) [104], [105]. Genes were considered differentially expressed if the FDR were <0.01. In addition, only genes with a signal log ratio of more than one or less than minus one were considered for further analysis.

### Gene Set Enrichment Analysis of DNA microarray results

To understand the biological significance underlying the gene expression data, gene set enrichment analysis (GSEA) was used [106]. This method analyzes all of the gene expression data to identify genes coordinately regulated in predefined gene sets. GSEA was applied independently to gene expression results obtained at 15 hpi and to those obtained at 65 hpi. Gene expression results were sorted by their logRatios. Gene Sets based on Gene Ontology keywords as defined in the subset C5 of Molecular Signatures Database (MSigDB v2.5) [106] were used. 1402 Gene Sets containing more than 4 and less than 501 members were considered. 1000 permutations were performed. In each case, the top 20 Gene Sets showing positive correlation with upregulated genes in our data were further analyzed.

### RNA analysis by qRT-PCR

Total RNA from Vero E6, or MA-104-infected cells was extracted using the Qiagen RNeasy kit according to the manufacturer's instructions and used to determine N gene subgenomic (sg) mRNA and genomic RNA levels by qRT-PCR. Reactions were performed at 37°C for 2 h with a High Capacity cDNA transcription kit (Applied Biosystems) using 100 ng of total RNA and the antisense primers Q-NsgSARS-RS (5′-TGGGTCCACCAAATGTAATGC-3′), complementary to nt 44 to 64 of N gene; and Q-SARS-2015-RS (5′- ATGGCGTCGACAAGACGTAAT-3′), complementary to nt 1995 to 2015 of genomic RNA. cDNAs were amplified by PCR using the Power SYBR Green PCR Master Mix (Applied Biosystems) and oligonucleotides Q-NsgSARS-VS (5′-AAGCAACCAACCTCGATCTC-3′), complementary to the virus leader sequence, and Q-SARS-1931-VS (5′-ACCACTCAATTCCTGATTTGCA-3′), complementary to nucleotides 1931 to 1952 of genomic RNA, and the oligonucleotides RS previously described [1]. All the primers were designed using Primer Express software (Applied Biosystems). Data were acquired with an ABI PRISM 7000 sequence detection system (Applied Biosystems) and analyzed with ABI PRISM 7000 SDS version 1.0 software. Levels of viral RNAs are represented in comparison to reference levels from cells infected with rSARS-CoV at 0 hpi.

For qRT-PCR of cellular genes, total RNA from Vero E6, and MA-104-infected cells was extracted as described above. Reactions were performed at 37°C for 2 h using a High Capacity cDNA transcription kit (Applied Biosystems) using 100 ng of total RNA and random hexamer oligonucleotides. Cellular gene expression was analyzed using TaqMan gene expression assays (Applied Biosystems) specific for human or monkey genes (Table 1). Data were acquired with an ABI PRISM 7000 sequence detection system (Applied Biosystems) and analyzed with ABI PRISM 7000 SDS version 1.0 software. Gene expression in rSARS-CoV-ΔE and rSARS-CoV-infected cells were compared. Alternatively, gene expression in rSARS-CoV-ΔE or SARS-CoV-infected cells was compared to mock-infected cells. Quantification was achieved using the 2^−ΔΔCt^ method, which is a convenient way to analyze relative changes in gene expression in qPCR experiments [107]. The data represent the average of three independent experiments.

**Table 1:** Taqman assays used to analyze the expression of cellular genes by quantitative RT-PCR.

Gene name	Taqman assay*	Description
DNAJA1	hs0266011-m1	DnaJ (Hsp40) homolog, subfamily A, member 1
DNAJA4	hs00388055-m1	DnaJ (Hsp40) homolog, subfamily A, member 4
DNAJB1	hs00428680-m1	DnaJ (Hsp40) homolog, subfamily B, member 1
DNAJB4	hs00199826-m1	DnaJ (Hsp40) homolog, subfamily B, member 4
DNAJB6	hs00369717-m1	DnaJ (Hsp40) homolog, subfamily B, member 6
DNAJC3	hs00534483-m1	DnaJ (Hsp40) homolog, subfamily C, member 3
SERPINH1	hs01060397-g1	serpin peptidase inhibitor, clade H (heat shock protein 47), member 1
hspA1A	hs00271229-s1	heat shock 70 kDa protein 1A
hspA1B	hs01040501-sH	heat shock 70 kDa protein 1B
hspA5/GRP78	hs99999174-m1	heat shock 70 kDa protein 5 (glucose-regulated protein, 78kDa)
hspA8	hs00852842-gH	heat shock 70 kDa protein 8
hspA9B	hs00269818-m1	heat shock 70 kDa protein 9B, mortalin
hsp90AA1	rh02791406-gH	heat shock protein 90 kDa alpha (cytosolic), class A member 1
hsp90AB1	hs00607336-gH	heat shock protein 90 kDa alpha (cytosolic), class B member 1
hsp90B1/GRP94	hs00427665-g1	heat shock protein 90 kDa beta (glucose-regulated protein, 94 KDa)
hspB1	hs03044127-g1	heat shock 27 kDa protein 1
hspD1	hs01036746-g1	heat shock 60 kDa protein 1 (chaperonin)
hspE1	hs00950982-gH	heat shock 10 kDa protein 1 (chaperonin 10)
hspH1	hs00971475-m1	heat shock 105 kDa/110 kDa protein 1
UBB	hs00430290-m1	ubiquitin B
UBC	hs01871556_s1	ubiquitin C
CCT3	hs00195623-m1	chaperonin containing TCP1, subunit 3 (gamma)
CCT4	hs00272345-m1	chaperonin containing TCP1, subunit 4 (delta)
BAG3	hs00188713-m1	BCL2-associated athanogene 3
AHSA1	hs00201602-m1	AHA1, activator of heat shock 90 kDa protein ATPase homolog 1 (yeast)
CRYAB	hs00157107-m1	crystallin, alpha B
18S	hs99999901-s1	Ribosomic RNA 18S
polQ	Hs00198196-m1	DNA polymerase, theta
TNF	Mm00443258-m1	Tumor necrosis factor
CCL2/MCP-1	Mm00441242-m1	Monocyte chemotactic protein 1
CCL5/RANTES	Mm01302428-m1	Regulated upon Activation, Normal T-cell Expressed, and Secreted
CXCL1/NAP-3	Mm04207460-m1	Neutrophil activating protein 3
CXCL2/MIP-2	Mm00436450-m1	Macrophage inflammatory protein 2
CXCL10/IP-10	Mm00445235-m1	Interferon inducible protein 10
IL-1α	Mm00439620-m1	Interleukin 1α
IL-1β	Mm01336189-m1	Interleukin 1β
IL-6	Mm00446190-m1	Interleukin 6
IFNγ	Mm01168134-m1	Interferon γ

*hs, means *homo sapiens*. rh, means rhesus (*Macaca mulatta*). Mm, means *Mus musculus*.

### Transfection of pcDNA3.1-E and infection with rSARS-CoV

Vero E6 cells grown to 90% confluence in M24 wells, were infected at an moi of 0.5 with rSARS-CoV-ΔE-P1 and -P16 and rSARS-CoV. Ninety min after infection, cells were transfected with 1 µg of the plasmid pcDNA3.1-E expressing the SARS-CoV E protein [77], or empty plasmid as control, using 1 µg of Lipofectamine 2000 (Invitrogen) according to manufacturer's instructions. Total RNA from mock- infected or rSARS-CoV-infected cultures was extracted at different times pi as described above and used to quantify the expression of the stress-response genes hspA1A, hsp90AA1, hspH1, SERPINH1 and hspE1 by qRT-PCR as described.

### Transfection of pcDNA3.1-E and infection with RSV

Vero E6 cells grown to 90% confluence in M24 multiwell plates were transfected with 1 µg of the plasmid pcDNA3.1-E, or empty plasmid as control, using 1 µg of Lipofectamine 2000 (Invitrogen) according to the manufacturer's instructions. After an incubation period of 5 h at 37°C, the transfection media were replaced and cells were incubated at 37°C for 24 h. Then, the cells were infected at an moi of 2 with RSV, Long strain [108]. RSV was provided by Dr. Blanca Garcia-Barreno (National Institute of Microbiology, Madrid), and titrated on Hep-2 cells as previously described [109]. Total RNA from mock-infected or RSV-infected cultures was extracted at different times pi as described above and used to quantify the expression of the stress-response genes hspAA1, UBB, hspH1, SERPINH1 and hspE1 by qRT-PCR as described.

### Transfection of pcDNA3.1-E and treatment with thapsigargin and tunicamycin

Vero E6 and MA-104 cells were transfected with plasmid pcDNA3.1-E or empty plasmid as above. Twenty-four hpt, cells were cultured in media containing 1000 nM thapsigargin, or 2 µg/ml of tunicamycin and incubated for another 8 or 20 hours, before analysis of expression of the UPR-induced genes, GRP78 and GRP94.

### Western blotting

Cell lysates were analyzed by sodium dodecyl sulfate-polyacrylamide gel electrophoresis (SDS-PAGE). Proteins were transferred to a nitrocellulose membrane with a Bio-Rad mini protean II electroblotting apparatus at 150 mA for 2 h in 25 mM Tris-192 mM glycine buffer, pH 8.3, containing 20% methanol. Membranes were blocked for 1 h with 5% dried skim milk in TBS (20 mM Tris-HCl, pH 7.5, 150 mM NaCl) and incubated with antibodies specific for hsp60 (Cell Signaling, Ref. 4870), hsp90 (Cell Signaling, Ref. 4877), SARS-CoV E protein (kindly provided by Shen Shuo, Institute of Molecular and Cellular Biology, Singapore), phospho-PERK (Santa Cruz Biotechnology, Ref. sc-32577), GAPDH (Abcam, Ref. ab9485), and ATF-6 (Abcam, Refs. ab11909 and ab37149). Bound antibodies were detected with horseradish peroxidase-conjugated goat anti-rabbit or anti-mouse antibodies (Cappel) and the ECL detection system (Amersham Pharmacia Biotech).

### RT-PCR analysis of XBP-1 mRNA

Total RNA from mock-infected or rSARS-CoV or rSARS-CoV-ΔE-infected cells was used for RT-PCR analysis of XBP-1 mRNA. cDNA was prepared using the specific oligonucleotide XBP1-RS (5′-CTGGGTCCTTCTGGGTAGAC-3′). cDNAs were amplified by PCR using the sense primer XBP1-VS (5′-CTGGAACAGCAAGTGGTAGA-3′), and XBP1-RS, flanking the splicing region of XBP-1 mRNA [69]. The RT-PCR products were resolved by electrophoresis in 2% agarose gels.

### Analysis of apoptosis in rSARS-CoV-infected cells

Vero E6 cells were grown to confluence in 12.5 cm^2^ flasks and infected at an moi of 4 with rSARS-CoV or rSARS-CoV-ΔE. At 4, 15 and 24 hpi, cells were treated with fluorescein isothiocyanate (FITC)-conjugated annexin V (Southern Biotech) to identify apoptotic cells measured by flow cytometry, as previously described [110]. Cells were then treated with 1 volume of 4% paraformaldehyde in PBS to inactivate virus. At the end of the process, propidium iodide (PI) staining was performed to differentiate cells in early apoptosis (Annexin V^+^, PI^−^) from those in late apoptosis (Annexin V^+^, PI^+^) stage.

## Supporting Information

Figure S1 **Cellular stress genes with expression levels similarly modified in rSARS-CoV-ΔE and rSARS-CoV infected cells versus mock-infected cells.** The differential expression of stress genes in rSARS-CoV-ΔE (X axis) and rSARS-CoV (Y axis) infected cells versus mock infected Vero E6 cells (blue symbols) and MA-104 cells (red symbols) studied using microarrays is represented. Symbol numbers correspond to the following genes: 1, CIP29; 2, DNAJC19; 3, DNAJA2; 4, DNAJC10; 5, hspA9; 6, DNAJC7; 7, hspA14; 8, DNAJB14; 9, DNAJB12; 10, hspA4; 11, DNAJC18; 12, DNAJC8; 13, DNAJC13; 14, DNAJC6; 15, DNAJC3; 16, DNAJC1; 17, DNAJB5; 18, hsp90B1; 19, DNAJB13.
(TIF)

Figure S2 **Cellular stress responses induced by rSARS-CoV-ΔE infection.** Vero E6 (A) and MA-104 (B) cells were infected with rSARS-CoV-ΔE or rSARS-CoV at an moi of 2. Cellular RNAs were extracted at 15, 22 and 28 (A) and at 24, 48, 65 and 75 (B) hpi, and the expression of cellular mRNAs corresponding to cytosolic, ER and mitochondrial stress was measured by qRT-PCR. Numbers indicate the level of gene expression in rSARS-CoV-ΔE compared to rSARS-CoV-infected cells. Three independent experiments were analyzed with similar results in all cases. Two commonly used acronyms of each protein are indicated at the bottom of the figure.
(TIF)

Figure S3 **Effect of S607F mutation in S protein on cellular stress responses induced by rSARS-CoV-ΔE infection.** Vero E6 cells were infected with the viruses lacking E gene passaged one or sixteen times (rSARS-CoV-ΔE-p1 and -p16, respectively) or with rSARS-CoV at an moi of 0.5. Cellular RNAs were extracted at 22 hpi and the expression of cellular mRNAs corresponding to cytosolic, ER and mitochondrial stress genes was measured by qRT-PCR. Numbers indicate the levels of gene expression in rSARS-CoV-ΔE-p1 or -p16-infected cells compared to rSARS-CoV-infected cells. Three independent experiments were analyzed with similar results in all cases. Two commonly used acronyms of each protein are indicated at the bottom of the figure.
(TIF)

Figure S4 **Effect of SARS-CoV E protein on the stress induced by infection with SARS-CoV.** Vero E6 cells infected at an moi of 0.5 with rSARS-CoV-ΔE-P16, or with SARS-CoV, were transfected with a plasmid expressing E protein (E^+^) or with empty plasmid (E^−^) as a control. At 22 hpi, cellular RNAs were extracted, and the expression of the stress-induced genes hsp10 A1A, hsp90 AA1, hsp H1, SERPIN H1, and hsp10 E1, and that of polQ and 18S rRNA, as controls, was analyzed by qRT-PCR. In each case, the corresponding mRNA expression levels in rSARS-CoV-ΔE-P16-infected cells were compared to those of rSARS-CoV-infected cells. Standard bars represent standard deviations of the mean of results from three experiments.
(TIF)

Figure S5 **Effect of SARS-CoV E protein on PERK activation.** Vero E6 cells were infected at an moi of 2 with rSARS-CoV-ΔE and rSARS-CoV. Cell extracts were collected at different times post-infection and the levels of the phosphorylated form of PERK, and of GAPDH as a reference control protein were analyzed by Western blot with antibodies specific for these proteins. pPERK levels in rSARS-CoV-ΔE or rSARS-CoV-infected cells, related to the levels of the housekeeping gene GAPDH are shown.
(TIF)

Table S1 **Level of cell infection by rSARS-CoV.** Human, porcine or monkey cells were infected at different mois, and the percentage of infected cells was measured by analyzing the presence of SARS-CoV N protein by immunofluorescence.
(DOC)
