# Supplementary figures and images for: Severe Acute Respiratory Syndrome Coronavirus Envelope Protein Regulates Cell Stress Response and Apoptosis
Source: PLoS Pathog. 2011 Oct 20;7(10):e1002315. doi: 10.1371/journal.ppat.1002315 (PMC3197621; doi:10.1371/journal.ppat.1002315)

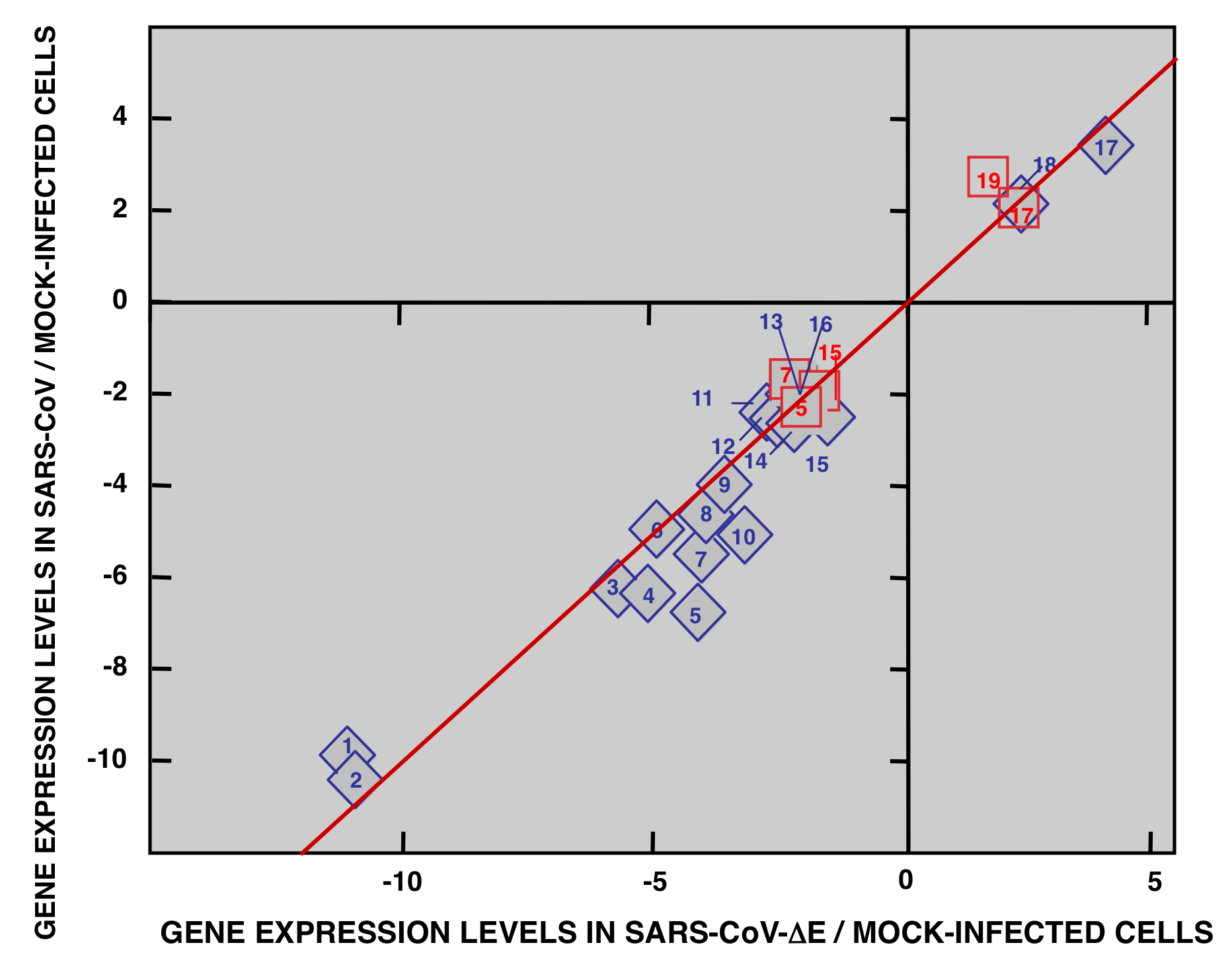

Supplement: Figure S1 — Cellular stress genes with expression levels similarly modified in rSARS-CoV-ΔE and rSARS-CoV infected cells versus mock-infected cells. The differential expression of stress genes in rSARS-CoV-ΔE (X axis) and rSARS-CoV (Y axis) infected cells versus mock infected Vero E6 cells (blue symbols) and MA-104 cells (red symbols) studied using microarrays is represented. Symbol numbers correspond to the following genes: 1, CIP29; 2, DNAJC19; 3, DNAJA2; 4, DNAJC10; 5, hspA9; 6, DNAJC7; 7, hspA14; 8, DNAJB14; 9, DNAJB12; 10, hspA4; 11, DNAJC18; 12, DNAJC8; 13, DNAJC13; 14, DNAJC6; 15, DNAJC3; 16, DNAJC1; 17, DNAJB5; 18, hsp90B1; 19, DNAJB13. (TIF) [file ppat.1002315.s001.tif]

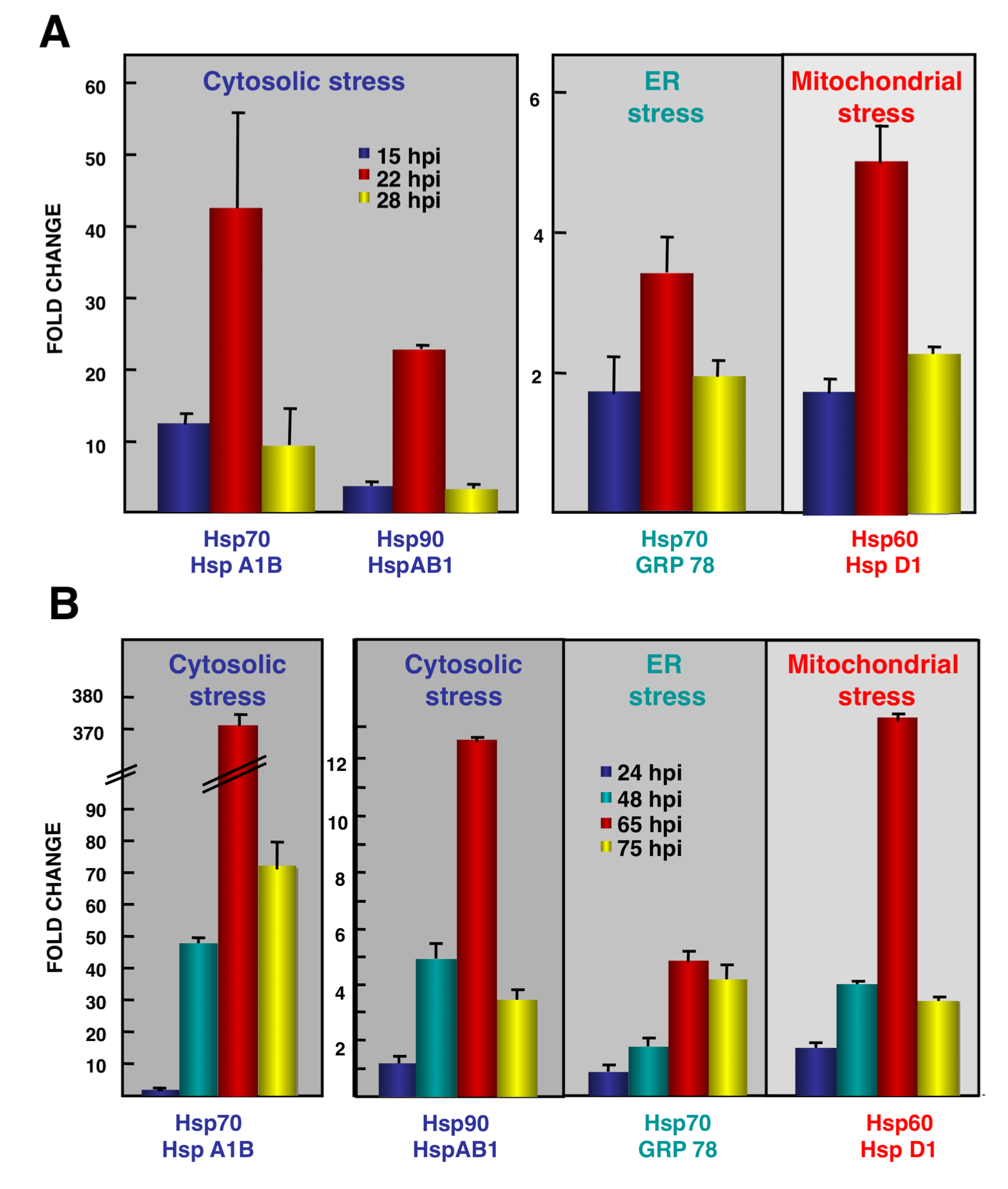

Supplement: Figure S2 — Cellular stress responses induced by rSARS-CoV-ΔE infection. Vero E6 (A) and MA-104 (B) cells were infected with rSARS-CoV-ΔE or rSARS-CoV at an moi of 2. Cellular RNAs were extracted at 15, 22 and 28 (A) and at 24, 48, 65 and 75 (B) hpi, and the expression of cellular mRNAs corresponding to cytosolic, ER and mitochondrial stress was measured by qRT-PCR. Numbers indicate the level of gene expression in rSARS-CoV-ΔE compared to rSARS-CoV-infected cells. Three independent experiments were analyzed with similar results in all cases. Two commonly used acronyms of each protein are indicated at the bottom of the figure. (TIF) [file ppat.1002315.s002.tif]

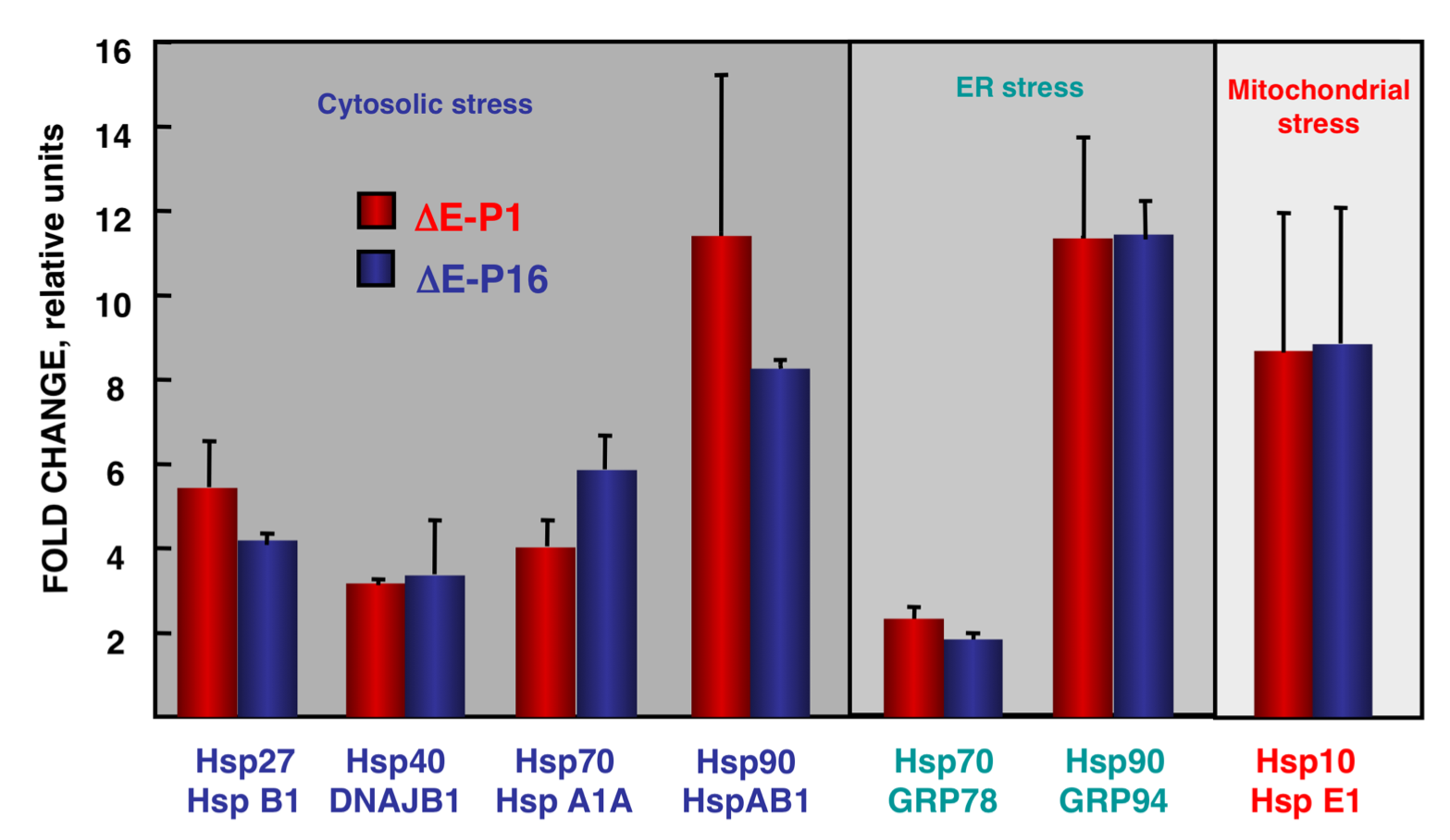

Supplement: Figure S3 — Effect of S607F mutation in S protein on cellular stress responses induced by rSARS-CoV-ΔE infection. Vero E6 cells were infected with the viruses lacking E gene passaged one or sixteen times (rSARS-CoV-ΔE-p1 and -p16, respectively) or with rSARS-CoV at an moi of 0.5. Cellular RNAs were extracted at 22 hpi and the expression of cellular mRNAs corresponding to cytosolic, ER and mitochondrial stress genes was measured by qRT-PCR. Numbers indicate the levels of gene expression in rSARS-CoV-ΔE-p1 or -p16-infected cells compared to rSARS-CoV-infected cells. Three independent experiments were analyzed with similar results in all cases. Two commonly used acronyms of each protein are indicated at the bottom of the figure. (TIF) [file ppat.1002315.s003.tif]

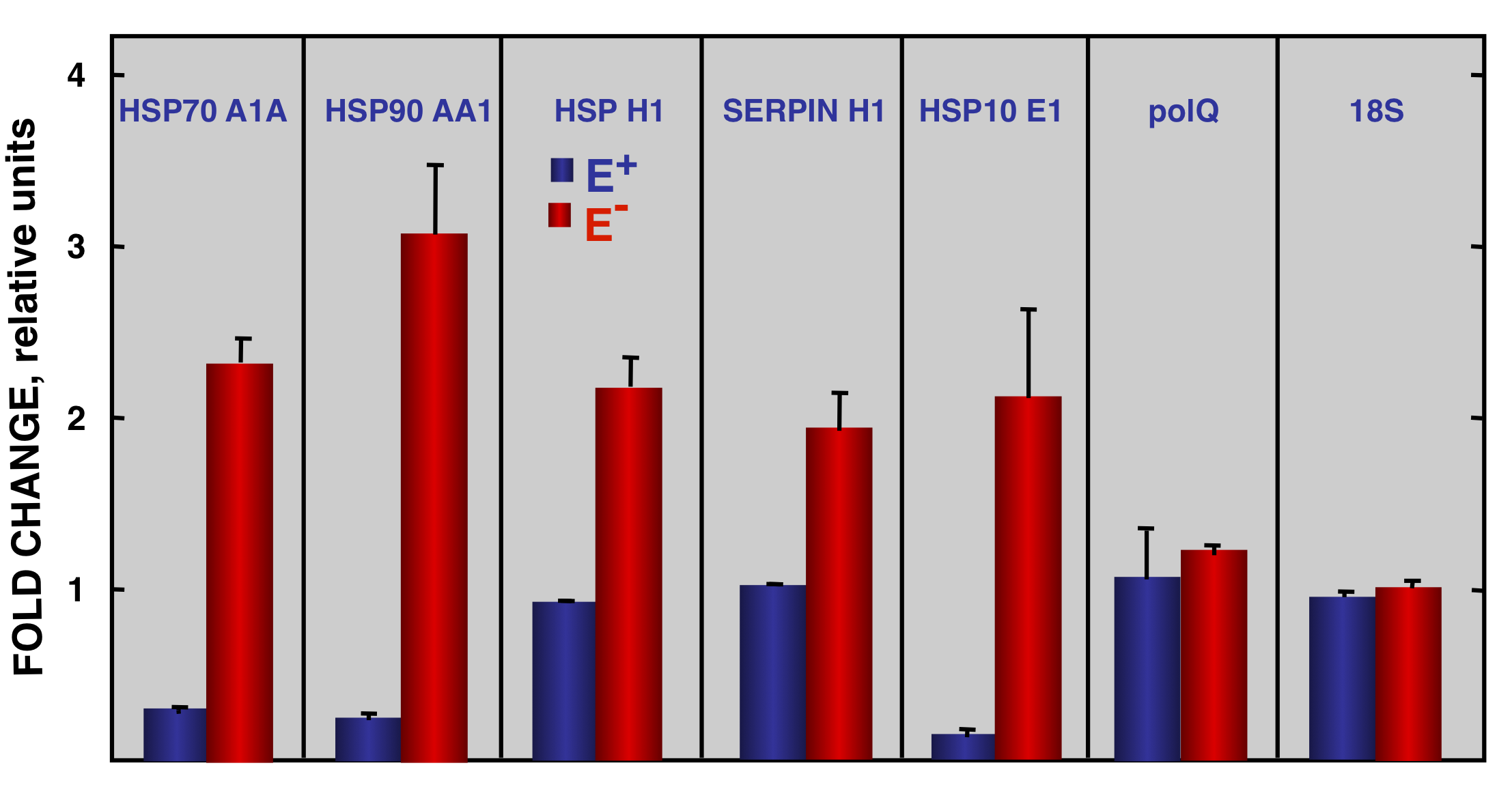

Supplement: Figure S4 — Effect of SARS-CoV E protein on the stress induced by infection with SARS-CoV. Vero E6 cells infected at an moi of 0.5 with rSARS-CoV-ΔE-P16, or with SARS-CoV, were transfected with a plasmid expressing E protein (E+) or with empty plasmid (E−) as a control. At 22 hpi, cellular RNAs were extracted, and the expression of the stress-induced genes hsp10 A1A, hsp90 AA1, hsp H1, SERPIN H1, and hsp10 E1, and that of polQ and 18S rRNA, as controls, was analyzed by qRT-PCR. In each case, the corresponding mRNA expression levels in rSARS-CoV-ΔE-P16-infected cells were compared to those of rSARS-CoV-infected cells. Standard bars represent standard deviations of the mean of results from three experiments. (TIF) [file ppat.1002315.s004.tif]

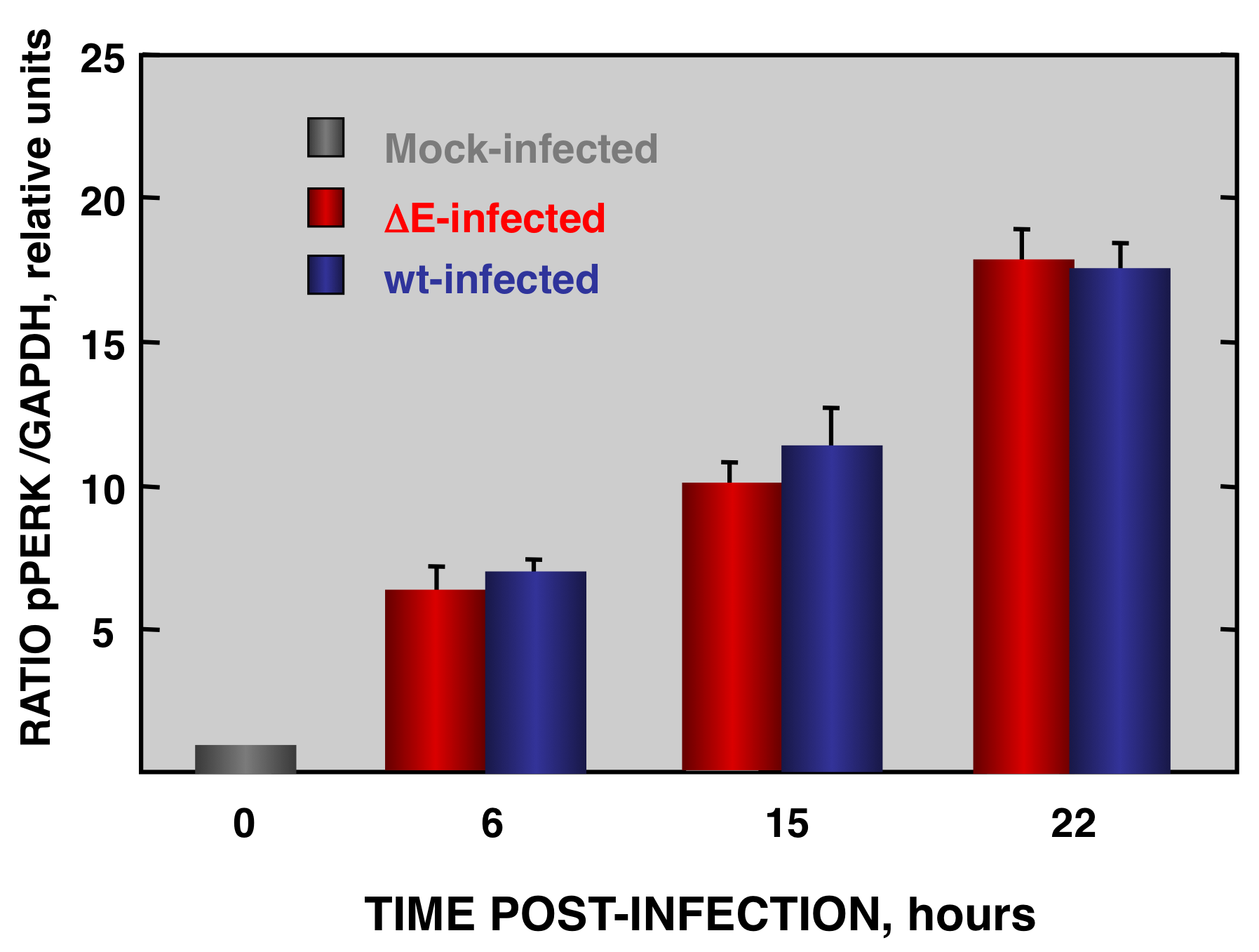

Supplement: Figure S5 — Effect of SARS-CoV E protein on PERK activation. Vero E6 cells were infected at an moi of 2 with rSARS-CoV-ΔE and rSARS-CoV. Cell extracts were collected at different times post-infection and the levels of the phosphorylated form of PERK, and of GAPDH as a reference control protein were analyzed by Western blot with antibodies specific for these proteins. pPERK levels in rSARS-CoV-ΔE or rSARS-CoV-infected cells, related to the levels of the housekeeping gene GAPDH are shown. (TIF) [file ppat.1002315.s005.tif]
