# Supplementary material for: Severe Acute Respiratory Syndrome Coronavirus Envelope Protein Regulates Cell Stress Response and Apoptosis
Source: PLoS Pathog. 2011 Oct 20;7(10):e1002315. doi: 10.1371/journal.ppat.1002315 (PMC3197621; doi:10.1371/journal.ppat.1002315)
Supplement: Table S1 — Level of cell infection by rSARS-CoV. Human, porcine or monkey cells were infected at different mois, and the percentage of infected cells was measured by analyzing the presence of SARS-CoV N protein by immunofluorescence. (DOC) [file ppat.1002315.s006.doc]

**SUPPLEMENTARY TABLE S1.** **Levels of infection in rSARS-CoV-infected cells**

| Cell line | **Infected cells*, %** | | |
| --- | --- | --- | --- |
|  | **Moi 1** | **Moi 3** | **Moi 5** |
| **Human cells** |  |  |  |
| HepG2 | <1 | <1 | ND |
| CaCo-2 | 1 | 2.5 | ND |
| Huh7 | 1.5 | 4 | ND |
| 293T | <1 | <1 | ND |
| 293 | ND | ND | 40 |
| **Pig cells** |  |  |  |
| PK15 | <1 | <1 | ND |
| **Monkey cells** |  |  |  |
| FRhK-4 | 5 | 15 | ND |
| MA-104 | ND | ND | >80 |
| Vero E6 | 20 | 53 | >90 |

*Cells were infected at mois 1, 3, or 5. At 24 hpi the percentage of infected cells was measured by analyzing the presence of SARS-CoV N protein by immunofluorescence. The percentage of infected cells in each case is shown. ND, not determined.
